# Accuracy, linearity, and statistical differences in comparative quantification in untargeted plant metabolomics using LC-ESI-Orbitrap-MS

**DOI:** 10.1007/s00216-025-05818-y

**Published:** 2025-03-10

**Authors:** Christina Maisl, Rainer Schuhmacher, Christoph Bueschl

**Affiliations:** Department of Agrobiotechnology IFA-Tulln, Institute of Bioanalytics and Agro-Metabolomics, BOKU University, Vienna, Tulln 3430 Austria

**Keywords:** Untargeted metabolomics, Accuracy, Linearity, Comparative quantification, Plant metabolomics, Orbitrap

## Abstract

High-resolution mass spectrometers, particularly when paired with liquid chromatography, are the instrument of choice for untargeted metabolomics approaches. Instruments, such as the Orbitrap, offer high sensitivity, selectivity, and exceptional mass accuracy, though they pose certain technical challenges, complicating absolute and comparative quantification. Consequently, method validation is crucial to ensure reliable results, as untargeted metabolomics approaches require the detection and quantification of a large number of metabolites in a broad dynamic range. Methods can be assessed using performance characteristics like accuracy and linearity to ensure analytical reliability. This study evaluates the suitability of untargeted metabolomics methods for discovery-based investigations. A stable isotope–assisted strategy was used with wheat extracts analyzed by a Q Exactive HF Orbitrap. Results showed that 70% of all detected 1327 metabolites displayed non-linear effects in at least one of the nine dilution levels employed. However, when considering fewer levels, 47% of all metabolites demonstrated linear behavior in at least four levels (i.e., a difference factor of 8). Moreover, the analysis further suggests that the observed abundances in less concentrated samples and those outside the linear range were mostly overestimated compared to expected abundances, but hardly ever underestimated. Consequently, during statistical analysis, which is an important step in prioritizing detected metabolites and correlating them with the biological hypothesis, the number of false-positives was not inflated, but the number of false-negatives might be increased. Generally, (non-)linear behavior did not correlate with specific compound classes or polarity, suggesting non-linearity is not easily predictable based on chemical structures.

**Graphical Abstract:**

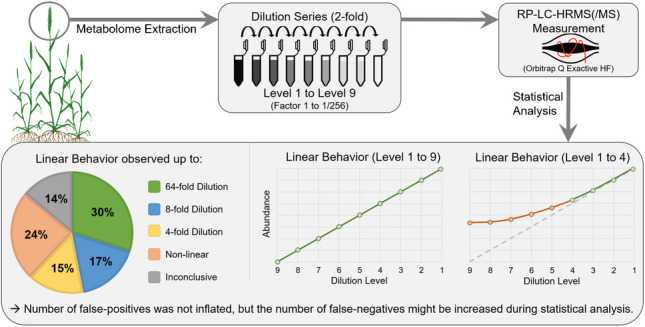

**Supplementary Information:**

The online version contains supplementary material available at 10.1007/s00216-025-05818-y.

## Introduction

Liquid chromatography mass spectrometry (LC-MS) is a frequently used technique in various fields of chemical analysis such as drug discovery, food, environmental, medical and bioanalytical, and also (untargeted) metabolomics [1–3]. One of the most commonly used mass spectrometer types are Orbitrap systems, which have proved to be robust, also for untargeted metabolomics approaches. The instruments are commonly used in combination with liquid chromatography and an electrospray ion source for untargeted approaches [4–6]. However, LC-MS systems (and biological sample matrices) are complex; thus, their method requires validation to ensure reliable results [1], especially when the aim is to compare different experimental conditions for differing metabolite levels.

The performance of an analytical method in respect of relative quantification can be evaluated with indicators such as accuracy (precision and trueness), linearity and linear range, limit of detection, limit of quantification, robustness, selectivity, and specificity to ensure that the applied analytical methods are actually fit for purpose [7]. In this context, the term linearity refers to the relationship between the concentration and detected signal of an analyte [8].

High-resolution mass spectrometers, such as Orbitrap systems, typically have high-resolving power, sensitivity, selectivity, and high mass accuracy [5, 9–11]. Coupled to ESI, which is a soft ionization technique, a large variety of different chemical compound classes can be ionized [12, 13], as long as the chemical species are transferred to gas phase and carry a functional group [12, 14]. However, untargeted metabolomics approaches not only require the detection of a large number of metabolites with a broad linear dynamic range, but also, that the measured abundances correspond to their relative concentration for comparison across different experimental groups or samples [15]. Still, all mass spectrometers including ESI-Orbitraps suffer from technical limitations (e.g., saturation effects during electrospray ionization or ion detection, ion suppression or loss of analytes by adsorption), which de- or increase relative abundances thereby complicating accurate relative quantification and as a result limit comparison of experimental groups [5, 15–17]. This means, concentration ranges, in which quantification is accurately possible, are limited and outside the respective linear range signal intensities are inaccurate (e.g., lost in noise or saturated at high intensity values) [16]. Additionally, comparative quantification based on EIC peak areas can be challenging as matrix effects and linearity can vary from compound to compound [15, 17–19]. Generally, the observed response in electrospray can be influenced by sample matrix, solvent (e.g., electrolyte concentration, and solution properties of the electrospray droplets), ionization conditions or LC-MS system components as well as matrix to analyte concentration ratio, compound concentration, mass, and charge of analytes, or pH of the chromatographic eluents [11–13, 20–23]. The term “matrix effects” commonly refers to an alteration of ionization efficiency in the ion source of the mass spectrometer caused by the presence of coeluting substances through competition for charges between an analyte and undetected (coeluting) matrix components. This can have a significant impact on the limit of detection/quantification and signal-to-noise ratio as well as linearity and accuracy [5, 18, 20, 21]. This competition for charges in the ion source can lead to ion suppression, potentially causing underestimation or non-detection of compound abundance, or overestimation by increasing the efficiency of ion formation [5, 18, 20]. Therefore, differences in the ionization efficiency of a particular compound result in varying response factors [13].

For targeted methods aiming at absolute quantification of selected metabolites, there are many studies investigating matrix effects or ion suppression specifically [5, 11, 18–22, 24–26] and how to assess (e.g., by linear regression, post-column infusion method, post-extraction spike method, dilution series experiment, internal standards) and/or mitigated/account for (e.g., by selective extraction strategies, sample clean-up methods, optimization of LC and/or MS parameters, “dilute-and-shoot” approach, strict data evaluation protocols, metabolic ratio correction) [4, 8, 18, 20, 27–32]. Each strategy has its own requirements and drawbacks, and it has to be evaluated individually which approach is the most effective for each case [28]. There are also many publications summarizing analytical method validation and strategies to improve performance parameters to which the interested reader is referred to [1, 7, 33–36]. However, to the best of the authors’ knowledge for untargeted metabolomics approaches there is a lack of studies of the accuracy and linearity.

To study linearity and accuracy of a workflow for untargeted plant metabolomics, a stable isotope–assisted approach was employed in here, to investigate accuracy and linearity for comparative quantification. The stable isotope–assisted approach not only enabled to detect only truly plant-derived compounds [37, 38], but also can be helpful in identifying and correcting matrix effects, as both the native and labelled compounds experience the same ion suppression or enhancement effects, with the response ratio of labelled to native compounds remaining the same [8, 25, 39–41]. At the example of wheat ear extracts analyzed with an untargeted metabolomics workflow employing an QExactive HF Orbitrap with an ESI source, a dilution approach was used to evaluate the relationship between analyte concentration and intensity of the detected MS signal in an untargeted way based on correlated signal response [30]. With this approach, we aimed to investigate:

- How large is the percentage of metabolites showing non-linear effects?
- How accurate can observed quantification differences be translated to true concentration differences for statistical interpretation?
- Are non-linear effects associated with chemical structure?
- Is there a general intensity cutoff value that can be used to estimate the limit of detection (LOD)?

## Materials and methods

### Chemicals and plant material

Frozen U-^13^C-labelled ears of Remus wheat cultivars were prepared as described in detail by Ceranic and colleagues [42] and briefly in “Cultivation and preparation of plants” section. Native Remus plants were kindly provided by Bernhard Seidl (University of Natural Resources and Life Sciences, Vienna, Department of Agrobiotechnology IFA-Tulln, Institute of Bioanalytics and Agro-Metabolomics, Konrad-Lorenz-Str. 20, 3430 Tulln, Austria). Details on plant cultivation and sampling can be found in Doppler et al. [43] and in “Cultivation and preparation of plants” section.

LC-grade methanol (MeOH) and acetonitrile (ACN) were purchased from Riedel de Haen, Honeywell (LC-MS grade, >99.9% purity, Seelze, Germany). MS-grade formic acid (FA) was purchased from Sigma-Aldrich (Vienna, Austria). ELGA water was obtained from an ELGA Purelab Ultra-AN-MK2 system (Veolia Water; Vienna, Austria).

For identification of metabolites, the following authentic reference standards were used: l-isoleucine, l-leucine, 5′-deoxy-5′-(methylthio)adenosine, guanosine, chlorogenic acid, glutathione, and cytosine (reduced form) (all from Sigma-Aldrich, Vienna, Austria) as well as adenosine (Molekula, Darlington, England), schaftoside and nicotinic acid (both from PhytoLab, Vestenbergsgreuth, Germany), and azelaic acid (Fluka, Vienna, Austria).

### Experimental setup

#### Overview

**Fig. 1:**
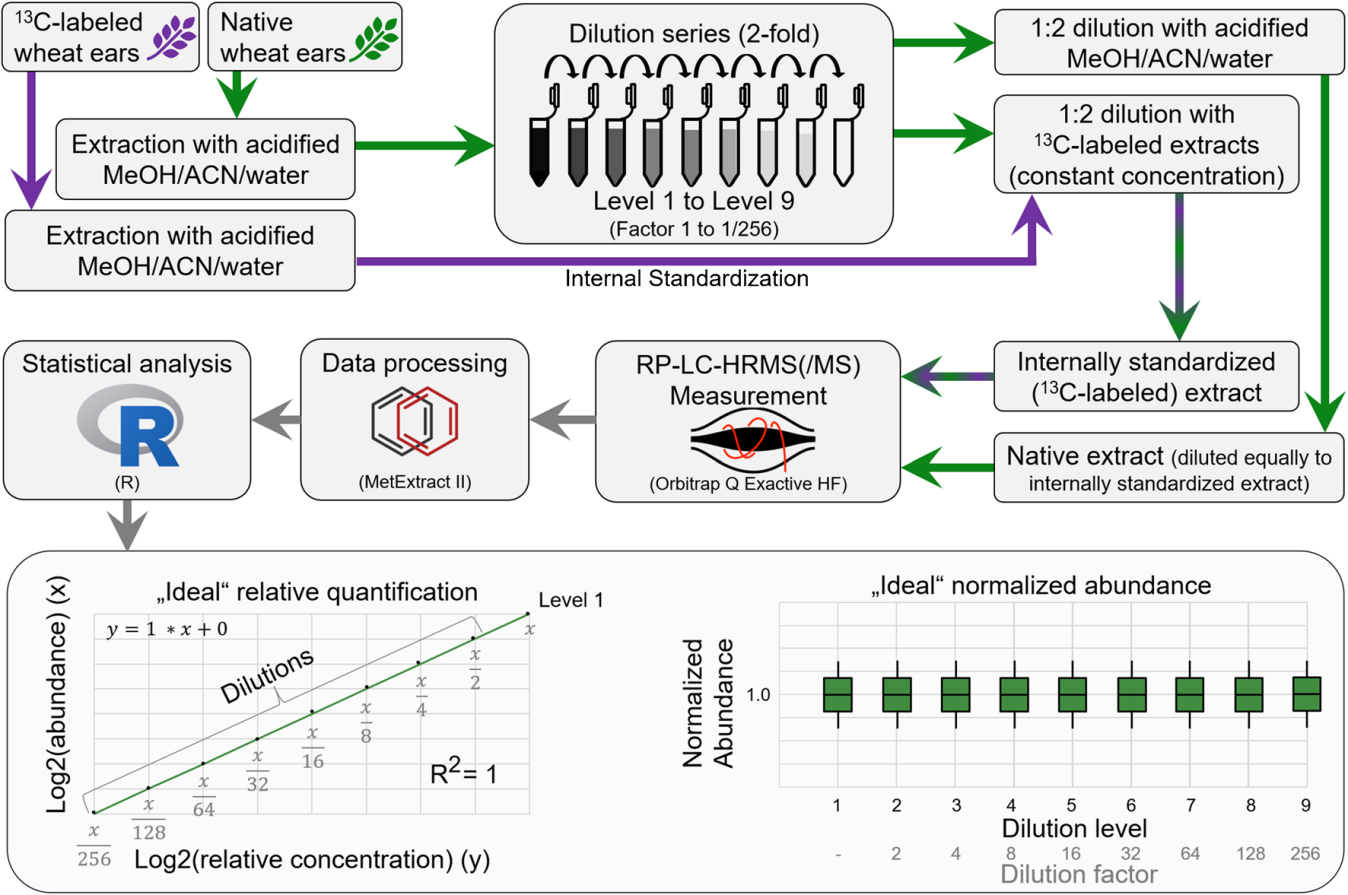
Overview of the experimental setup showing also examples of “ideal” results at the example of linear regression and illustration of response factors

An overview of the experimental setup is shown in Fig. 1.

Native and labelled wheat ears were extracted with a solvent mix (“Metabolome extraction” section). A dilution series was established (2-fold per step, 8 dilutions) of the native extracts. The dilution series was made three times, always starting from a separate extract of native wheat ears, and each series was subsequently split into two aliquots, of which one was diluted with solvent, while the second aliquot was diluted with an extract of the ^13^C-reference. The concentration of labelled extract was kept constant across all dilutions of native extracts for metabolome- and experiment-wide internal standardization. This way, the native and labelled dilution series exhibited similar concentrations and are comparable. Each sample was then measured three times using RP-LC-HRMS, data was processed with MetExtract II, and statistical analysis was carried out in R. If no non-linear effects occurred, then the results would show an “ideal” linear regression (Fig. 1).

It should be noted here that the experimental design was similar to our previous study investigating the performance of lyophilization as sample preparation technique in untargeted plant metabolomics [44].

#### Internal standardization with stable isotopically labelled plant material

Using a well-established workflow that allows extracting only pairs of native and ^13^C-labelled metabolite ions, it is possible to put the focus on only the true plant metabolites by filtering out any unspecific features resulting from background compounds (i.e., non-wheat-metabolites such as plasticizers, contaminants of solvent) [37, 38, 44]. The detailed workflow and protocols for plant cultivation and handling are described by Céranic and colleagues [25] and in “Cultivation and preparation of plants” section. By keeping levels of labelled metabolites constant across all dilutions of native extracts, the extract of labelled plants serves as internal standardization and enables comparison across all experimental groups [39, 45].

### Sample preparation

#### Cultivation and preparation of plants

Native and ^13^C-labelled Remus plants at their flowering stage were utilized. The handling of the labelled plants is described by Céranic and colleagues [42]. Briefly summarized: ^13^C labelling of wheat plants was performed in an air-tight growth chamber (phytolabelbox), where environmental conditions were controlled and enriched ^13^CO_2_ was used. Seeds were germinated, vernalized, transferred into the phytolabelbox, and cultured as described by Céranic and colleagues [42]. At the flowering stage, plants were harvested and ears were cut off and immediately frozen in liquid nitrogen. Labelled plant ears were lyophilized at −80 °C to reduce water content below 5% (FreeZone 6 Plus, Labconco, Kansas City, MO, USA) and stored at −80 °C until further sample preparation [44].

Native plants were cultivated in the glass house without ^13^CO_2_ and harvested at the flowering stage as described by Céranic and colleagues [25]. Briefly summarized, the plant ears were cut off and immediately frozen in liquid nitrogen.

Native fresh and labelled lyophilized ears were stored at −80°C until further sample preparation.

#### Metabolome extraction

Ears were milled to a fine powder (MM301 Retsch, Haan, Germany; 30 Hz for 60 s), which was stored at −80 °C until extraction. Extraction was carried out according to the protocol of Doppler and colleagues [46], similar to our previous study [44]. Native and labelled ears were extracted separately. To increase the concentration of extracted compounds, instead of a ratio of 1:10 (homogenized plant ears to extraction solvent), a ratio of 1:5 was used. This means, for fresh native ears, per 100 mg of homogenized ears, 500 µL of pre-cooled extraction solvent (MeOH/ACN 1 + 1 (v/v) mixed with water (3 + 1, v/v) + 0.1% formic acid) was used. For lyophilized labelled ears, 30 mg of homogenized ears was mixed with 70 µL of pre-cooled ELGA water to achieve a water content similar to fresh ears as well as 500 µL of pre-cooled extraction solvent (MeOH/ACN 1 + 1 (v/v) mixed with water (3 + 1, v/v) + 0.1% formic acid).

Extracts were vortexed for 10 s and put into an ultrasonic bath (47 kHz, 105 W) for 15 min. Next, extracts were centrifuged (14,000 rpm, 4 °C) for 10 min. Supernatants were transferred into a fresh test tube. Organic solvent content was reduced to 50% (v/v) by adding water + 0.1% formic acid. Extracts were then vortexed for 10 s and centrifuged again (14,000 rpm, 4 °C) for 10 min. The supernatants were transferred into HPLC vials for LC-HRMS measurement. In total, three native extracts were prepared for three separate dilution series. Process blanks were prepared accordingly by using water instead of plant extracts.

#### Dilution series

Three dilution series of extracts of native wheat ears were prepared by repeated 2-fold dilution with a mixture of MeOH/ACN/H_2_O 1:1:2 + 0.1% FA. In total, eight dilution levels were prepared.

Then, of each dilution series sample, an aliquot was taken and mixed with ^13^C-labelled wheat ear extract to keep the concentration of labelled extract constant over the whole dilution series for the purpose of internal standardization. In a pre-experiment, the ratio of native to labelled extract was tested to have approximately similar native and labelled content. Results showed that in this case a ratio of 1:2 (native to labelled) showed approximately equal content (data not shown).

Another aliquot of the dilution series of native ears was diluted accordingly with MeOH/ACN/H_2_O 1:1:2 + 0.1% FA for comparison.

### LC-HRMS analysis

The extracts were analyzed, similar to our previous study [44], using an UHPLC system (Vanquish) coupled to an Orbitrap Q Exactive HF mass spectrometer (Thermo Scientific, Bremen, Germany) equipped with a heated electrospray ionization source. For chromatographic separation, a C18 reversed-phase column (3.5 μm; 2.1 mm × 150 mm; XBridge^®^; Waters; Milford, MA, USA) was used. Autosampler temperature was set to 10 °C and the column temperature was set to 25 °C. For each sample, 2 µL was injected. Each sample will be measured three times (technical replicates). The sequence order of samples was randomized, to avoid instrumental drift that affects the results [33]. Water containing 0.1% formic acid (eluent A) and methanol containing 0.1% formic acid (eluent B) were used as eluents. A linear gradient with a constant flow of 250 µL/min was used for elution starting with 10% eluent B and continuously increasing B to 100% at 10 min after an initial hold time of 1 min. After a hold time of 3 min, the column was re-equilibrated for 7 min at 10% eluent B. Full scan MS measurements were acquired with fast polarity switching to generate positively and negatively charged ions with a scan range of *m/z* 80 to 1200 and a resolution of 120,000 FWHM. The auxiliary and sheet gas flow rates were set to 5 and 55 units, respectively. Spray voltage was set to 3500 V for positive and 3000 V for negative mode. MS/MS measurements of original (undiluted) extracts were performed in a positive and negative mode separately with a scan range of *m/z* 100 to 1000 and a resolution of 30,000 FWHM. Collision energies were set to 20, 45, and 70 eV stepped.

### Data processing and statistical analysis

#### Data processing and metabolite annotation

LC-HRMS raw data files were converted to the mzXML format using MSConvert of ProteoWizard (v3.0.21292) and subsequently processed with MetExtract II [37] (parameter settings are provided in Supplementary Information 1). Inclusion lists for targeted MS/MS spectra acquisition were generated from the detected features of undiluted samples. MS/MS measurements were processed with MZmine 3 (parameter settings are provided in Supplementary Information 2), matched to MetExtract II output, and exported to the mgf format for metabolite annotation and molecular networking. Compound classes were predicted with CANOPUS [47] (SIRIUS, v5.5.7). XCalibur Qual Browser (v4.2) and FreeStyle were used to compare detected metabolites with MS/MS measurements of in-house standards for annotation. Level 1 annotation (identified) required matches to standards with retention time shift less than 10 s and principal ions shifted by less than 5 ppm *m/z*.

#### Statistical analysis

Statistical analysis was carried out in R (https://r-project.org, v3.5.3). Further information about the used methods and parameters are provided in Supplementary Information 3.

#### Normalization of data

To improve depiction and account for large differences in the peak areas across different metabolites, the abundance of each feature was normalized. This was done for each feature and sample independently by dividing the abundances of a feature by the mean value of the abundance of the particular feature in only level 1 (i.e., undiluted sample). This is referred to as “level 1 normalized abundance.”

Furthermore, to better illustrate differences between different dilution levels, another adjustment method was used, where the normalized abundances of the features found for the different dilution levels were multiplied by their respective dilution factors relative to level 1 (i.e., abundance measured for level 1 was multiplied by 1, that of level 2 was multiplied by 2, that of level 3 was multiplied by 4, etc.). This is referred to as “dilution-adjusted abundance.”

Comparison of coefficients *k* and *d* of linear models for metabolites: To compare differences in linear regression between the different clusters, the coefficients *k* and *d* of generated linear models (*y* = *k* * *x* + *d*) were calculated for each metabolite in the three measurements separately. In order to compare the coefficients of the models, normalized abundances were used. Thus, an ideal linear regression where the metabolite’s relative abundance changes with the dilution level and is not affected by any other effect has the coefficients *y* = 1 * *x* + 0 (*k* = 1 and *d* = 0). Any regression close to this optimal coefficient has a linear response in the dataset, while stronger deviations from this optimum indicate non-linear behavior.

A lack of fit test was employed to test if a quadratic model approximates the dilution series better than a linear model. Furthermore, the maximum range of dilution levels a metabolite is linear in was established by full enumeration, meaning that for any two dilution levels and all within those (e.g., levels 2 and 5 as well as 3 and 4) a linear model and a lack of fit test was calculated. Then, the maximum linear range for a metabolite was defined as those levels where the linear model resulted in an *R*^2^ value of at least 0.95 and the lack of fit test did not report that a quadratic model approximated the abundances better than a linear model.

Volcano plots use dilution-adjusted abundances of the native (monoisotopic ^12^C) features. Therefore, in case of ideal linear regression, all metabolites should be positioned at a fold-change of 1 and at a log2-fold-change of 0, respectively. Therefore, if metabolites show linear behavior, it is expected to see no features, which significant differences respectively between the different levels because they were properly normalized by calculation of dilution-adjusted abundances.

## Results

### Data overview

#### Suitability of data

To ensure that the extracts used in this study are suitable to investigate the performance of technical aspects of the untargeted metabolomics workflow under observation, it was verified that the extracts cover a broad, representative range of metabolites and compound classes. Overall, 1327 true wheat-metabolites from 6632 ions were detected. Of these metabolites, 470 were detected as ions in the positive and negative ionization mode, while another 360 and 497 metabolites were only detected as ions in the positive or negative ionization modes, respectively. Furthermore, the detected features covered a broad range of *m/z* values and the entire retention time range (Fig. S1a) as well as a large intensity range which—as expected—decreases with increasing dilution level (Fig. S1b) for both positive and negative ionization modes.

Subsequently, 1009 (positive mode) and 956 (negative mode) precursor ions were subjected to MS/MS fragmentation in separate measurements. Metabolite annotation was performed using CANOPUS [47], which revealed different compound (super) classes, such as fatty acids and conjugates, amino sugars and aminoglycosides, flavonoids, glycerolipids, monoterpenoids, phenolic acids, small peptides, and many more. Thus, with this strategy, many different compound classes were detected and about 25% of all metabolites (i.e., 357) were annotated.

For a general overview, a principal component analysis was performed (Fig. S2). Samples of the same dilution level cluster together, while a distinct separation between samples differing in dilution levels can be observed, as expected. Moreover, a stronger separation of those levels with higher metabolite abundances (levels 1 and 2) is evident already in the first principal component.

In summary, with the large number of diverse metabolites, the utilized wheat ear extracts are deemed suitable for this technical study.

#### Accuracy and linearity

Accuracy of analytical measurements consists of two components: trueness and precision. Absolute trueness is not accessible for a sample with unknown analyte content [33]; however, in the here diluted extracts, we can make use of the dilution levels and the thereby generated expected abundance differences between two levels (e.g., multiplication of feature abundance of the diluted extracts with the respective dilution factor). This is shown in “Linearity and trueness of relative quantification” section and “Examples of representative metabolites” section. Precision, however, can be shown in different ways and here it was chosen to use relative standard deviation (RSD). Here, this measure comprises all steps in the experimental pipeline, i.e., sample preparation, analytical measurements, as well as data processing (e.g., peak picking and integration).

Overall, RSD values were on median below 15% for native and 10% for labelled extracts throughout all experimental groups of the dilution series (Fig. S3). Similar, however partially slightly increasing in stronger diluted extracts, RSD values between dilution levels were observed, thus suggesting that repeatability and precision are high in the dataset, comparable to what has been observed previously and much lower than typical biological variability [37, 38].

**Fig. 2:**
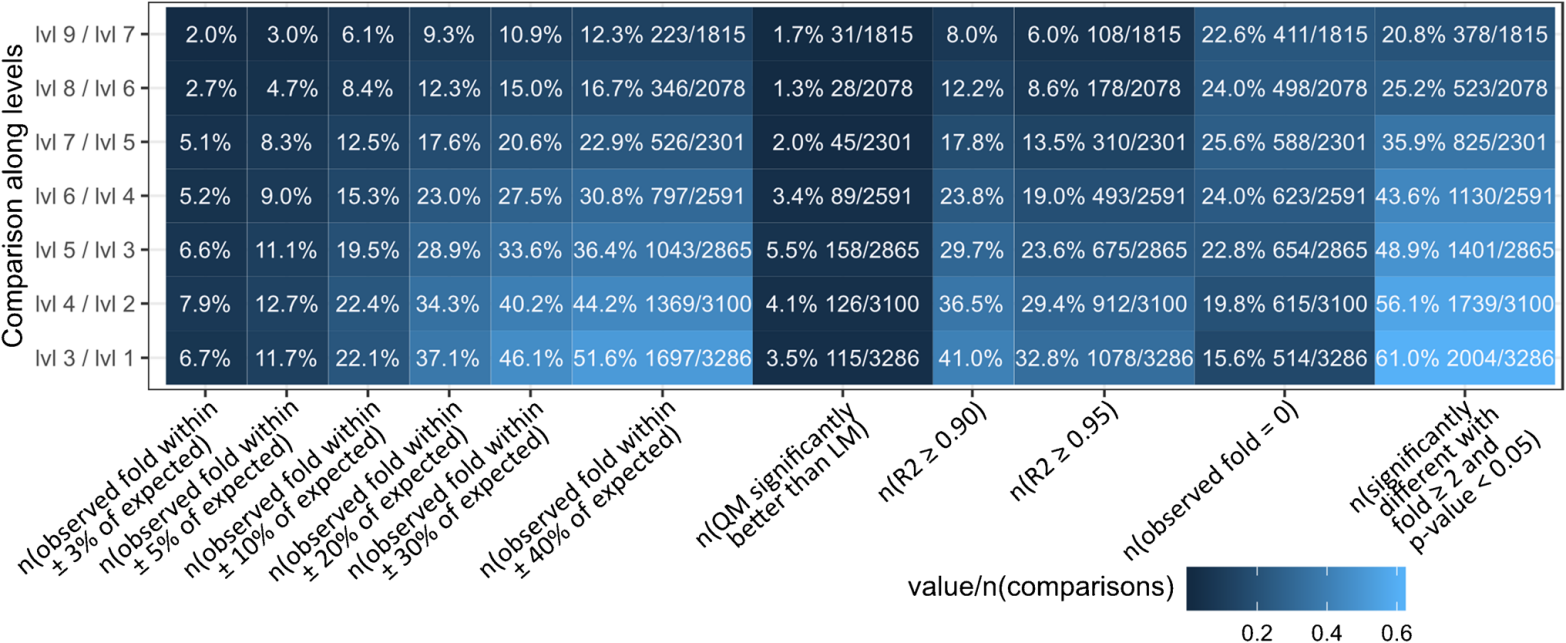
Overview of linear model performances over three dilution levels (Fig. S4 shows all dilution levels)

When comparing measured with expected abundances or their fold-change between dilution levels, it is expected to, for example, see a 50% decrease of abundance between directly adjacent levels, e.g., level 2 vs. 1, level 3 vs. 2, or only 0.25 (i.e., ¼) of the original abundance when comparing level 3 vs. 1, level 4 vs. 2, and so on. For example, the expected and observed fold-changes between levels 2 and 1 were within a 20% window for 52.7% of the metabolites (Fig. S4, columns “n(observed fold within ±XX% of expected fold)”). As expected, this value steadily decreased with increasing fold-changes, e.g., comparing level 3 to level 1 (undiluted) resulted in 37.1% (Fig. 2), level 4 to level 1 (Fig. S4) showed 27.6% up to level 9 to level 1 (undiluted) which showed 3.3% with less than 20% deviation. In case of 40% maximum tolerated deviation, 51.6% (levels 3 vs. 1) to 5.5% (levels 9 vs. 1) of metabolites showed the expected fold-change value.

Next, linear calibration curves were established for the detected metabolites across all tested levels. Using the determination coefficients (*R*^2^) to assess linearity can be misleading (data with curvature could also show high *R*^2^ values) [8], residuals from linear regression analysis can be investigated as a secondary criterion to confirm linearity [33]. According to SANCO/12571/2013 guideline for example, relative residuals below 20% are considered acceptable [33]. In case of the presented study, this criterion is fulfilled for the majority of metabolites (for most comparisons, more than 75% of metabolites show on average residuals below 20%); however, the more dilution levels were used for the linear models, the higher maximum values of the residuals became, which is to be expected (Fig. S5). Additionally, performing a lack of fit test, which is based on the analysis of residual variance, showed that a quadratic model was significantly better (Fig. S4, column “n(QM significantly better than LM)”) than a linear model for 1.3% (level 8 to level 6) up to 9.8% (level 9 to level 1) of metabolites. No clear correlation between the number of quadratic models performing better than linear models and included dilution levels or metabolite abundances seems to be present.

Therefore, overall, the use of linear models does not lead to systematic errors with respect to the resulting abundance for the majority of metabolites. However, not all metabolites showed the expected reduction of abundances with each dilution step, which could be reasoned with the presence of non-linear effects, which is investigated in the next chapters.

### Comparative quantification

#### The (almost) ideal example

The perfect method for untargeted metabolomics experiments has no matrix effects, interferences, saturation, data processing problems, or other non-linear effects and is also capable of accurately quantifying and identifying any metabolite in a sample (“Overview” section, Fig. 1). However, such a method does not exist (now). Current analytical methods suffer from the above limitations.

Therefore, if the presented dilution series were to be analyzed with the perfect, but only imaginary method, abundance values of a dilution level would always be half (in case of a 2-fold dilution) of the respective previous, more concentrated level. Furthermore, the respective linear model would report high *R*^2^ values. Additionally, when the metabolite abundances of each level are adjusted to their respective dilution level (by multiplication with their respective dilution factor) and normalized to the mean abundance observed for level 1 (“Normalization of data” section all values would be 1, as shown in the simulated plot for “ideal” normalized abundance (Fig. 1).

**Fig. 3:**
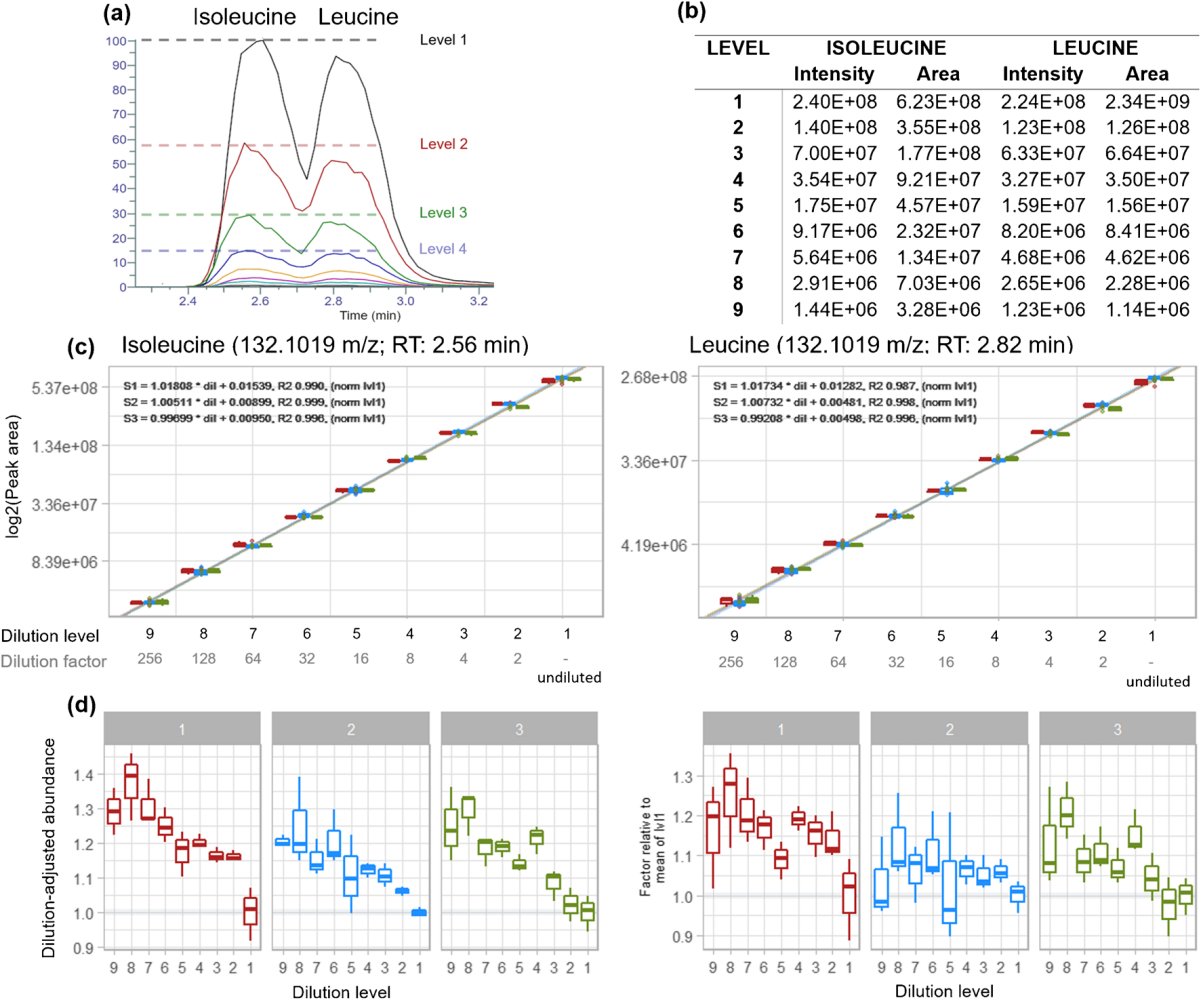
**a** EICs of measurement 1. **b** Intensity (peak height) and peak area values of leucine and isoleucine obtained from dilution series 1 measurement 1. **c** Linear regression and **d** normalized abundances (multiplied by dilution factor) relative to level 1 (undiluted) of isoleucine and leucine. The colors (red, blue, green) in (**c**) and (**d**) represent the three measurement sequences

In our study, the two compounds isoleucine and leucine reported high *R*^2^ values (compare Fig. 3), almost the ideal linear model coefficients (close to *y* = 1 * dil + 0.) and a dynamic range from 2.4E8 to 1.4E6 signal intensity (levels 1 to 9) for isoleucine and 2.2E8 to 1.2E6 for leucine (levels 1 to 9), with abundance values being approximately 50% less in consecutive dilution levels. Additionally, the dilution-adjusted abundances (Fig. 3d) show only low deviations with level 9 reaching a maximum deviation factor of approximately 1.4.

This almost ideal, linear behavior of leucine and isoleucine is unfortunately not observed for all metabolites. In the next chapter, statistical clustering techniques are utilized to find patterns among the linear models and deviations and to investigate if these correlate with chemical structures.

#### Holistic comparison

**Fig. 4:**
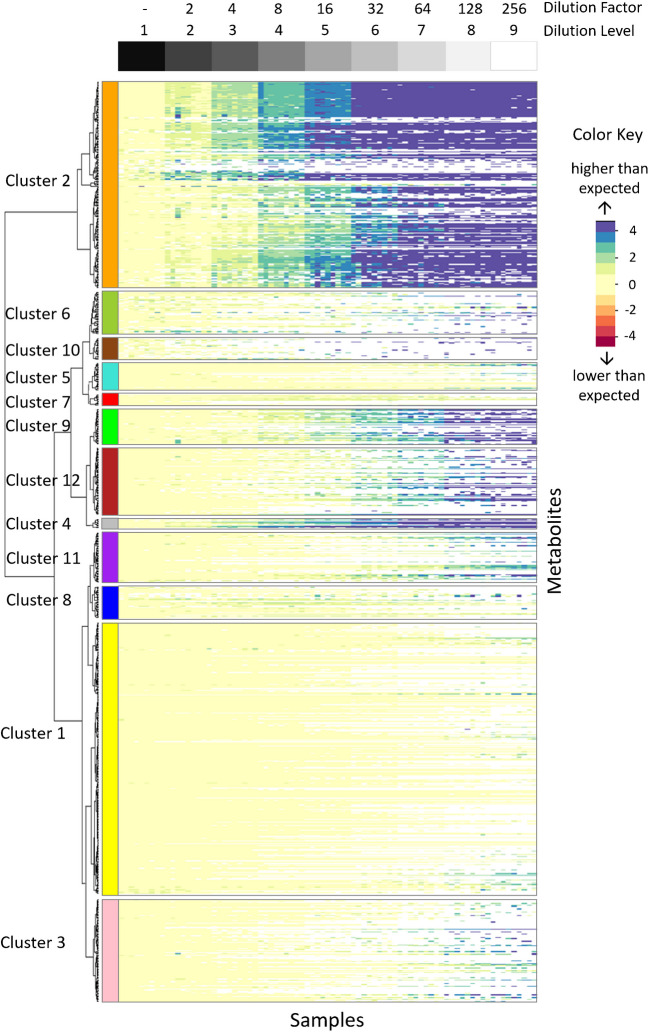
Heatmap illustrating the log2 values of dilution-adjusted abundances of native (monoisotopic ^12^C) features of internally standardized (not corrected) extract per dilution level. Yellow color indicates a dilution-adjusted abundance of approximately 1, while increasing blue color indicates increased abundances compared to the expected values. Deviations of dilution-adjusted abundances from 1 of an ideal linear relationship are capped at 4 for improved visualization

Next, the same evaluation strategy as for isoleucine and leucine was applied to all metabolites under investigation. For this, the dilution-adjusted abundances (“Normalization of data” section) of the monoisotopic ^12^C (native; not IS-corrected) metabolites were clustered by HCA across samples (Fig. 4), thereby putting metabolites with similar response factors through the dilution series in clusters. The resulting dendrogram was subsequently cut in a supervised manner into twelve clusters each containing metabolites with similar linear or non-linear behavior.

Overall, the generated heatmap shows that the first few dilution levels (i.e., with highest metabolite concentrations) did not deviate largely from the expected values. However, several metabolite clusters showed quite diverse patterns regarding their dilution-adjusted abundances throughout the dilution series. The largest cluster (cluster 1, yellow; 30% (301) of metabolites) consists mostly of well-behaving metabolites that are all close within the expected value of 1 (yellow color in the heatmap), which indicates linear behavior of the respective metabolites throughout all dilution levels. Furthermore, the two example metabolites leucine and isoleucine are also part of this cluster.

However, on the other hand, there are also metabolite clusters for which their dilution-adjusted abundance values deviate strongly from 1, e.g., the second largest cluster (cluster 2, orange; 23% (227) of metabolites). This deviation and blue-shaded values are indicative for non-linear effects. In cluster 2 (orange), the expected value of 1 and therefore a yellow color is observed only in the more concentrated samples of dilution levels 1 to 3, while the higher dilutions gradually resulted in increasingly strong deviations from that optimum. The other clusters are smaller and more diverse. For example, cluster 12 (firebrick) and cluster 9 (green) show yellow color in the heatmap until approximately dilution level 3, but onwards deviate more towards blue and increasingly darker colors. Generally, only a low number of red color keys are observed, but these rather seem to be isolated outliers. Furthermore, the number of metabolites that were not detected in a particular sample increased with the dilution level, which can be expected as metabolites concentrations start to be below the limit of quantification and detection (empty cells in the heatmap, shown in white).

In summary, metabolites affected by non-linear effects show increasingly differing abundances with increasing dilution factors (Fig. 4). Mostly, observed metabolite abundances were higher than expected, and only a minor number of abundance values were below the expected values. Furthermore, in Figs. 4 and S4 (columns n(observed fold within ±XX% of expected)), it is obvious that this deviation of the observed vs. expected abundance is highly level dependent and increases with each additional dilution level.

#### Influence of polarity, intensity, and structure of compounds on linearity

**Fig. 5:**
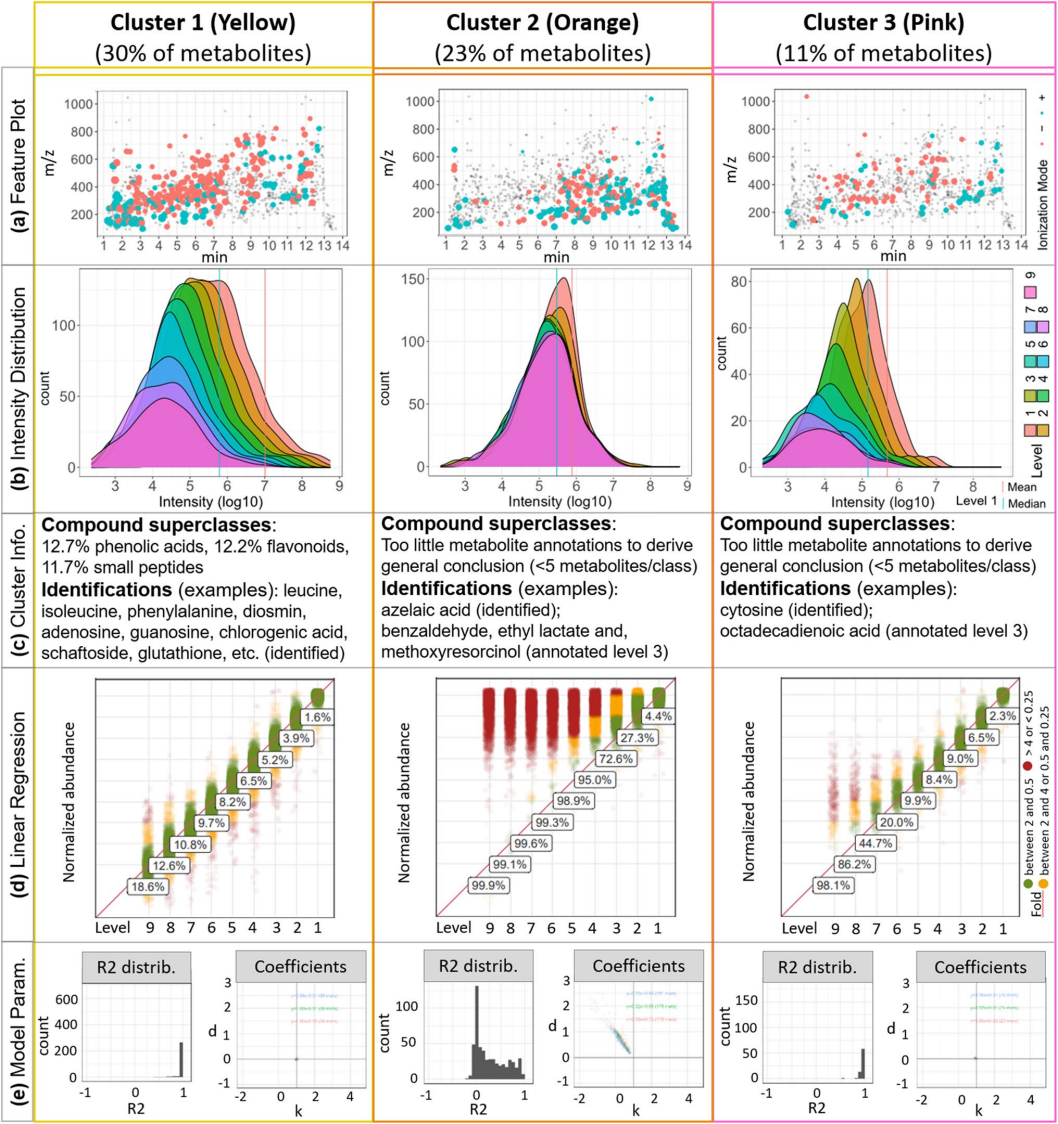
**a** Feature plot of the respective heatmap cluster shown in Fig. 4 (red, negative mode; blue, positive mode; gray, all detected features). **b** Distribution of peak apex intensity per level and metabolite cluster (red line, mean intensity of level 1; blue, median intensity of level 1). **c** Summary of chemical information derived from Sirius. **d** Plots of normalized abundances for each dilution level obtained for the metabolites assigned to the respective cluster (plots for all cluster are available in Fig. S9). Green color indicates metabolites with a fold-change between the 2 or 0.5 interval relative to the expected normalized abundance, yellow color indicates a fold-change between 2 and 4 or 0.5 and 0.25, and red color indicates a fold-change value greater than 4 or less than 0.25. Labels indicate the percentage of metabolites outside the green interval. **e** Parameters of linear models. Left plot: Histograms of *R*^2^ values. Right plot, overview of coefficients *k* (slope) and *d* (intercept). Plots for all subclusters from (**e**) are available in Figs. S11 and S12 respectively

Next, linear or non-linear behavior was correlated with different compound structure–related properties such as polarity, *m/z* values, and chemical classes. All clusters covered broad ranges of *m/z* values in both positive and negative ionization modes, as well as a wide range of polarities (i.e., retention time). No strong correlation was observed for these properties (*m/z* value and retention time; Figs. 5a and S6), except for cluster 7 (red). This cluster mainly consists of metabolites with early retention times between 1 and 5 min. However, as there are only 15 compounds in that cluster, a general conclusion cannot be drawn for these.

To test that the metabolite clusters (Fig. 4) were not formed based on higher or lower signal intensities, the peak apex intensities were illustrated as separate density plots for the respective metabolite cluster (Figs. 5b and S7). It could be expected that lower-abundant metabolites are generally more affected by non-linear effects; however, only minor differences were observed in the respective intensity distributions of the metabolites in the different clusters. Generally, intensity values range from 1E3 to 1E9 for the undiluted extracts (i.e., level 1). Clusters with high numbers of metabolites (e.g., clusters 1 (yellow), 2 (orange), and 3 (pink)) had a broad range of intensity values, with the median being approximately between 1E5 and 1E6. On the other hand, in clusters with a low number of metabolites (e.g., blue, red, gray, turquoise), the intensity values were much sparser, but also covered the entire intensity range. Cluster 1 (yellow) clearly showed a reduction in the average peak intensity across the different dilution levels and at higher dilution levels also a considerable reduction in the number of metabolites detected. Cluster 3 (pink) showed this decrease in the number of metabolites already at dilution level 3. On the other hand, cluster 2 (orange) showed only a reduction of the number of metabolites with increasing dilution level that flattens out at level 3 and then does not change any more. Cluster 4 (gray) showed barely any difference between the different dilution levels, which indicates false-positive detections or re-integration of noise (“Linearity and trueness of relative quantification” section).

Next, compound superclasses and pathways of metabolites underlying the respective cluster were annotated using SIRIUS (“Statistical analysis” section). Results of NPC and annotations of the three largest clusters are summarized in Figs. 5c and S8. Then, the (non-)linear behavior of metabolites was organized in a molecular network to correlate chemical structures with the assigned metabolite groups. However, no definite correlation between chemical properties and the established linearity clusters was observed (data not shown).

In summary, while there are minor differences in the metabolites assigned to the different sub-clusters, no strong correlation could be observed between the assigned clusters and mass, polarity, chemical structure or classes, and abundances.

#### Linearity and trueness of relative quantification

**Fig. 6:**
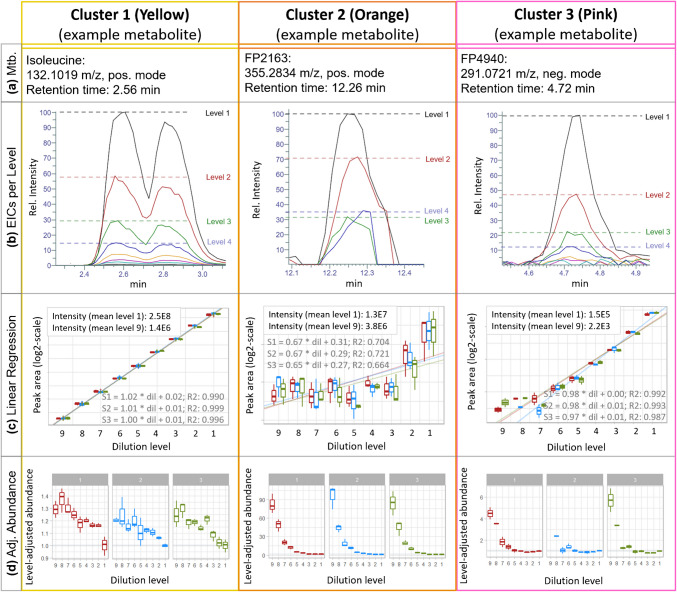
**a** Metabolite (Mtb.) information. **b** EICs of representative metabolites for each dilution level. **c** Linear regressions obtained for the examples. **d** Box plots showing the dilution-adjusted abundances of the selected metabolites

For a more detailed investigation of linear (or non-linear) behavior, the observed and expected normalized abundances were plotted for each metabolite cluster of Fig. 4 (Fig. 5d). The metabolites assigned to cluster 1 (yellow) showed the expected reduction of abundance in the dilution series and only few deviated from the expected values (Fig. 5d). Only after level 7 (which corresponds to a dilution of 1/64 compared to dilution level 1) more than 10% of the metabolites deviate more than a fold-change greater than 2 relative to the expected dilution factor. Therefore, comparative quantification of metabolites from this cluster is possible. On the other hand, metabolites of the two other cluster showed strong deviations from the expected values already in less diluted extracts; e.g., metabolites in cluster 3 (pink; 11%, 112 metabolites) start to deviate after level 5 (dilution factor of 1/16) from expected values, showing increasingly higher abundances (up to 4.5-fold for level 9). In strong contrast, the deviations from ideal linear regression in cluster 2 (orange; 23% of metabolites) were already observed in level 2. Additionally, the factor by which the metabolites of cluster 2 (orange) differ from ideal dilution-adjusted abundances increased with increasing dilution level (compare Volcano plots in Fig. S10), plateauing at a factor of 100 or higher (or even 600 for individual metabolites) for dilution level 9 (Fig. 5d; Fig. S9) rendering any comparison between the dilution levels incorrect. A potential explanation for this could be integration of noise during the re-integration step of the data processing workflow, which is the replacement of missing abundance or peak areas with integrated signal intensities due to missing true chromatographic peaks that are detected with the respective peak picking algorithm. However, this unavoidably leads to the integration of noise and alters linear regression drastically. Unfortunately, this cannot be solved by applying a filter based on a minimum signal-to-noise-ratio (SNR) threshold, as, for example, the example metabolite (Fig. 6) of cluster 2 (orange) showed high SNRs (>100; extracted from MetExtractII) even in diluted samples, and, generally, no clear correlation was observed between linear or non-linear metabolite behavior and SNR values. Additionally, SNR values can vary significantly between and within days [1] and also between measurements. Besides that, calculated SNRs vary greatly depending on the software or algorithm used and can e.g. have values exceeding 1E17 (FreeStyle, Thermo Scientific), which indicates issues with peak and/or noise detection. However, an extensive evaluation of the performance of different algorithms for re-integration is beyond the scope of this study and should be the subject of follow-up studies.

The distribution of the linear regression coefficients (“Normalization of data” section) for metabolites of cluster 1 (yellow) showed that most coefficients were close to their optimal and expected values (*k* = 1, *d* = 0, all levels used for linear models; Fig. 5e). These coefficients mean that the peak areas of most metabolites reduce by 50% for consecutive dilution levels and steps, which is optimal as it allows to easily calculate mean-fold-changes during statistical analysis. Interestingly, deviations from the optimal linear regression coefficients (up to *d* = 0.5 and *k* > 1) were present when calculated for dilution level 1 (undiluted) and 2 only. This could indicate signal saturation in undiluted and less diluted extracts. A similar effect, but with much stronger deviations (*k* down to −2 and *d* up to 3) is observed for cluster 2 (orange). Additionally, the (non-)linear behavior of the clusters is reflected in *R*^2^ value distribution (Fig. 5e). An overview of all linear regression coefficients in the different clusters is shown in Fig. S12.

Generally, it seems a general linear range can hardly be defined based on abundance distributions, as also indicated in “Influence of polarity, intensity, and structure of compounds on linearity” section An overview of the abundances in the highest and lowest levels is shown in Fig. S13. As expected, less diluted samples exhibit higher intensities for the metabolites. On the contrary, similar signal intensity distributions were observed across different linear models for highly diluted samples, regardless of the range covered (Fig. S13, y-axis), which indicates a range for an intensity cutoff below which metabolites are no longer reliably within their linear ranges. This range was between 1E4 to 1E6 (two orders of magnitude) and the 90% percentile for the lower intensity values is 2.4E5 in positive and 2.3E5 in negative mode. The intensity of 1E5 is exceeded for 78.8% in positive mode and 78.2% in negative mode.

In summary across all clusters, about 30% of all 1327 wheat-metabolites had linear behavior up to a 64-fold dilution, 47% showed linear behavior up to an 8-fold dilution, and 62% showed linear behavior up to a 4-fold dilution. On the other hand, 24% of the metabolites already showed non-linear behavior at a 2-fold dilution, and 14% showed inconclusive behavior. Moreover, a single intensity value at which the metabolites show linear behavior could not be found and it rather seems to be metabolite dependent. However, it was observed that 90% of abundances within the linear range have an intensity value above 2.3E5.

#### Examples of representative metabolites

Besides the cluster-based overview of linear behavior, a closer investigation was performed for some chosen metabolites (Fig. 6a).

The example of cluster 1 (yellow) showed that the abundance of isoleucine is approximately halved with each dilution step, as expected (Fig. 6b). This was also observed for the example of cluster 3 (pink) for the shown dilution levels (levels 1 to 4). The example metabolite of the orange cluster showed higher abundances (70%) than expected (50%) already after the first dilution (level 2), which indicates non-linear behavior. As already pointed out in section “Influence of polarity, intensity, and structure of compounds on linearity,” the abundances seem to not correlate with linear or non-linear behavior, which is also reflected on these examples, with the less abundant metabolite (of cluster 3 (pink)) showing linear behavior in contrast to the more abundant example metabolite (of cluster 2 (orange)). Additionally, with increasing dilution level, the peak of the example metabolite of cluster 2 (orange) is seemingly lost in noise, but reappears in the data matrix after re-integration (“Linearity and trueness of relative quantification” section). This is also observed for the example of cluster 3 (pink) below dilution level 4. The peak intensity reduced from 1.5E5 (level 1) to 7.9E3 (level 5), while noise level (observed in level 5) showed intensities of approximately 5E3. These results were also reflected in the linear regression plots (Fig. 6c), with high *R*^2^ values (0.996) for cluster 1 (yellow) and cluster 3 (pink) indicating linear behavior, while low *R*^2^ values (0.696) were observed for the example in cluster 2 (orange).

Ideally, if metabolites have linear behavior, the boxplots (Fig. 6d) show identical dilution-adjusted abundance in all dilution levels (i.e., all values are scattered around 1, Fig. 1, “Overview” section). A value of 2 therefore means that the measured value is twice as high as the expected value, and thus an overestimation of actual concentrations by the factor of 2. The example metabolite of cluster 1 (yellow) showed almost ideal linearity throughout all dilutions, and only a slight increase in normalized abundances with increasing dilution factor of up to 1.4 was observed for higher diluted levels (Fig. 6a). Cluster 3 (pink) showed ideal (linear) behavior up until dilution level 5. Differences to level 1 (undiluted) reach up to a factor of 6 for level 9, thereby overestimating the measured metabolite abundances. The example metabolite of cluster 2 (orange) showed a strong deviation from the ideal behavior, with dilution-adjusted abundances reaching deviation factors of up to 90 for level 9.

Overall, it was observed that there is a clear tendency towards non-linear effects and the observed differences increased for higher dilution levels, suggesting higher abundances of the compounds than truly present in the sample (Fig. 6d).

#### Intensity vs. area—a comparison on sample and metabolite level

In our analysis, we found that linear regression calculations can be effectively conducted using both peak areas and metabolite intensities. On both the sample and feature levels, the results garnered from using metabolite intensities and areas proved to be comparable, exhibiting a high correlation coefficient of 0.975, with only minor deviations observed (Fig. S14). Focusing on intensity values specifically below the defined linear range of the experiment (i.e., spanning from 1E4 to 1E6 as outlined in section “Linearity and trueness of relative quantification”), the correlation coefficients, on average, decreased to 0.873. This diminution in correlation was anticipated owing to the increased relative contribution of noise in lower intensity values, which can significantly deviate peak area estimations. A more thorough investigation into this phenomenon, however, extends beyond the scope of this study.

## Discussion

The 1327 detected metabolites covered a wide range of *m/z* values and polarity, showed high precision, and were assigned to many different compound classes including phenolics and fatty acids, which are two quite diverse and large groups (“Suitability of data” section). Consequently, it represents a fairly diverse plant metabolome and was deemed suitable for investigating linear effects and probe comparative quantification for untargeted metabolomics studies.

### Extent of non-linear effects

In an ideal world, untargeted metabolomics methods would not suffer from matrix effects, interferences, saturation, data processing challenges, and other non-linear effects, thus allowing accurate quantification and identification of all metabolites within a sample. However, no such method (currently) exists. Present analytical techniques are always constrained by these limitations to some extent. For example, electrospray ionization (ESI) of LC-HRMS measurements is prone to disturbances from coeluting sample components, which can lead to non-linear effects and reduced accuracy of compounds [5, 33]. Ion suppression or enhancement can occur in ESI, leading to a flat or steeper slope of linear regression respectively. Besides that, saturation of ionization can occur, which leads to a non-linear relationship at higher concentration. Moreover, compounds can be lost through adsorption which typically leads to a negative y-intercept, or interferences can lead to a positive y-intercept [21, 48].

The presented study showed that under the tested conditions, 70% of metabolites showed non-linear behavior in at least one of the dilution levels (Fig. 4) regardless of the measured intensity (Fig. 5). A similar trend was observed in a study of serial diluted urine samples, where only 12% of signal ratios were not compressed or inflated [31]. Additionally, non-linear effects reduce the statistical power of *t*-tests as shown by a study of Yu and colleagues [32].

A general linear range could hardly be defined as there is no clear correlation between linear behavior and the measured EIC peak height or area and (non-)linear behavior rather seems to be metabolite dependent. Still, on the lower end, results showed that the intensity of 2.3E5 seems to be the lower limit for 90% of the tested metabolites (“Linearity and trueness of relative quantification” section). Thus, peaks with intensity values below 2.3E5 should be considered to not exhibit linear behavior when performing comparative analysis or quantification, which could be reasoned in integration issues of low abundant peaks, integration of noise, etc. Though, it should be noted this threshold is highly instrument and method dependent, and the value itself cannot be translated directly to other methods. Furthermore, it is important to recognize that sample matrices can significantly influence the response of metabolites and their resulting (non-)linear behavior, due to potential interactions between compounds within those matrices.

The analysis of the dataset also indicates that only a small number of metabolites are better represented by a quadratic model; hence, a simpler linear model is applicable for the majority of the identified compounds. Furthermore, since the slope and intercept values for most metabolites are close to their anticipated figures (see Fig. S12), the statistical tests and effect size calculations can be conducted directly on the raw peak areas, which makes data analysis for most experiments much easier.

Furthermore, in the presented study, it was not possible to correlate linear or non-linear effects to certain compound classes, the polarity (via retention time), or structural features (“Influence of polarity, intensity, and structure of compounds on linearity” section). This indicates that, potentially, other, so far unidentified factors are involved. A possibility would be for example that compound classes are a too broad classification and rather the presence of certain chemical side-groups, which are not covered by the used annotation approach, are (partially) responsible for the observed effects.

Moreover, no clear correlation between linear or non-linear metabolite behavior and signal-to-noise ratios was observed (“Linearity and trueness of relative quantification” section).

### Lessons learned for comparative quantification and statistical analysis

One of the most commonly employed methods to investigate quantitative differences in untargeted metabolomics is univariate statistics volcano plots (Fig. S10), which illustrate *p*-values and a mean-fold-change of two experimental groups thereby directly comparing peak areas or intensity values. The fact that the presented analysis mostly reported over-estimated peak areas or intensity values in more strongly diluted samples (Fig. 4) affects the outcome and power of statistical analysis as well. This overestimation leads to lower mean fold-changes between the tested dilution levels compared to expected values, an observation that Yu and colleagues [31] also made in their study of human urine samples. Consequently, metabolites reported to be significantly differing according to a *p*-value (≤ 0.05) and a mean-fold-change (≥ 2) can be considered to have at least the observed fold-change. However, on the other hand, metabolite abundances for which statistical testing reports no significant difference might be affected by the non-linear relationship and thus are incorrectly not detected as a significantly different.

Therefore, the presented results suggest that the number of metabolites incorrectly classified as significantly differing between tested groups is not inflated, but the number of metabolites incorrectly classified as not statistically different might be increased. This is also seen in the presented experiment when the total number of possible comparisons yielding a fold of at least 4 are summarized. For 30.6% of these tests (any two levels and metabolite with the respective metabolite detected in at least one of the two levels; tests with the respective metabolite not detected in both groups were discarded; in total 79,082 comparisons) a significant difference was reported, which is quite low and probably not what is generally expected from untargeted approaches as this means that about 2/3 of all tests are incorrectly reported as not significantly different. Generally, the number of univariate tests reported as significantly different is higher when the metabolites are more abundant (e.g., the comparison of lowly diluted levels 3 to 1 (undiluted) reports 61.0% significantly different metabolites) than when their abundances are lower (e.g., the comparison of the strongly diluted levels 9 to 7 only reports 20.8% significantly different metabolites), which is what can be expected.

Despite the expectation that a higher proportion of tests would be correctly reported as significantly different, only about one-third met this criterion. However, this finding must be interpreted with caution. In addition to the tests classified as significantly different, another 36.6% of the univariate tests need to be considered separately. For those tests, the fold-change could not be determined as the respective metabolite was only detected in the group consisting of the less diluted samples, but not in the group with the more strongly diluted samples (i.e., metabolites below level of quantification/detection), thereby leading to an inconclusive fold-change of 0. For these tests it is difficult to state if the respective metabolite is different or not, especially if no other information (e.g., peak area or intensity) is utilized. However, such cases are expected and rather the norm than the exception, which also motivated the development of different re-integration steps in software tools (e.g., background signal or noise integration to replace missing values).

To correctly assess these tests, additional information is required to find those metabolites that are largely abundant in one group but not the other. It now no longer suffices to have a fold-change value between the two groups, but one must be certain that the abundances in the group where the respective metabolites was detected are much higher than the LOD. Therefore, knowing (or determining) a general or metabolite-dependent intensity cutoff can be helpful in these cases as well, as it can also serve as an additional criterion (e.g., an LOD-fold). For example, this new LOD-fold can be calculated between the detected abundances and this LOD. Then, similar to fold-changes, the LOD-fold can help in estimating if the metabolites exceed this LOD and therefore also the abundances in the group in which the respective metabolite was not detected. However, as shown earlier, it is difficult to estimate or calculate a general LOD value and thus such an analysis needs to be carefully evaluated to not introduce false results into the statistics. In general, such a strategy is already commonly used when the missing values are replaced by an LOD or LOD-half value. However, a detailed investigation of this issue was out of the scope of this manuscript.

In respect to re-integration, the presented results showed that a re-occurring problem with low abundant metabolites during data processing was that abundances were wrongly estimated due to re-integration of noise instead of actual metabolite signals (“Linearity and trueness of relative quantification” section). The integration of noise led to seemingly non-linear relationships in stronger diluted samples. However, re-integration can still be helpful to reduce missing values. However, to avoid the observed issue of noise integration an additional filtering step (based on an empirically estimated intensity cutoff through a dilution series; “Linearity and trueness of relative quantification” section) could be added in the data processing workflow to filter out all features below a certain intensity value, for example.

## Conclusion

The goal of this study was to estimate how much of the wheat metabolome is affected by non-linear effects, how accurate observed quantification differences can be translated to true concentration differences, if non-linear effects are associated with chemical structure, and if there is a general intensity cutoff value.

It can be concluded that non-linear behavior was observed for a large number of the detected metabolites (i.e., 70% of the 1327). Still, 62% of the detected metabolites showed linear behavior when only the less diluted samples (levels 1 to 3) were considered, meaning that these do not fail to report a difference of at least a fold-change of 4. Furthermore, for 47% of metabolites, a linear behavior was observed up to level 4, which confirms that untargeted metabolomics approaches are a strong and reliable tool for discovery-based experiments, but technical limitations in the form of heavily overestimated peak areas lead to a strongly increased number of metabolites, whose abundances are incorrectly not reported as significantly differing (inflated type II errors). Generally, linear behavior of metabolites could not be correlated with specific compound classes, measurement ionization mode or chemical polarity suggesting that linearity is independent of larger chemical compound classes and rather seems to be metabolite dependent.

The presented results also confirm the common assumption that results of comparative quantification and univariate statistical testing are more reliable for metabolites with abundances on the upper spectrum of the LC-HRMS instrument reported signal values. However, it should be considered that ion suppression can occur at high metabolite abundances. Moreover, a single, general intensity cutoff could not be defined. Thus, on the lower end, the 90% percentile was used as a surrogate for this threshold, which, for the used instrument and analytical method, is 2.3E5. Signals below that intensity might show inaccurate results in comparative experiments under the tested conditions. Therefore, to estimate the lower intensity value beneath which signals should be discarded and, additionally, to discard metabolites which do not show linear behavior if accurate fold-change values are of interest, a recommendation of the presented study is to include a dilution series in untargeted metabolomics experiments of a (pooled) sample to test for linear behavior and eventually discard metabolites that show non-linear effects to challenge comparative quantification and establish its limitations either on a per experiment or a per metabolite basis.

In summary, while comparative quantification and non-linear effects seem to affect a large portion of the metabolome, untargeted approaches also are powerful and are a reliable tool for discovery-based approaches to find statistically different metabolites. Moreover, the presented data suggests that metabolites reported as significantly different are truly different (low type I error), but that differences might not be detected and metabolite abundances might be incorrectly reported as not significantly differing between groups (inflated type II error).

## Supplementary Information

Below is the link to the electronic supplementary material.

Supplementary file1 (PDF 4430 KB)

## Data Availability

The data presented in this study are openly available in GNPS/MassIVE at (doi:10.25345/C57H1DZ6N), reference number MSV000095588.
