# Supplementary material for: Accuracy, linearity, and statistical differences in comparative quantification in untargeted plant metabolomics using LC-ESI-Orbitrap-MS
Source: Anal Bioanal Chem. 2025 Mar 10;417(11):2293–309. doi: 10.1007/s00216-025-05818-y (PMC11996957; doi:10.1007/s00216-025-05818-y)
Supplement: Supplementary file 1 — Supplementary file1 (PDF 4430 KB) [file 216_2025_5818_MOESM1_ESM.pdf]

Analytical and Bioanalytical Chemistry

Supplementary Information for

Accuracy, Linearity, and Statistical Differences in Comparative Quantification in Untargeted Plant Metabolomics using LC-ESI-Orbitrap-MS

Christina Maisl[1], Rainer Schuhmacher[1], Christoph Bueschl[1]

[1] BOKU University, Vienna, Department of Agrobiotechnology IFA-Tulln, Institute of Bioanalytics and Agro-Metabolomics, Konrad-Lorenz-Str. 20, 3430 Tulln, Austria

Table of Content

Fig. S1 2

Fig. S2 3

Fig. S3 4

Fig. S4 5

Fig. S5 6

Fig. S6 7

Fig. S7 8

Fig. S8 9

Fig. S9 10

Fig. S10 11

Fig. S11 12

Fig. S12 13

Fig. S13 14

Fig. S14 15

Data Processing parameters 16

  Supporting Information 1 16

  Supporting Information 2 16

  Supporting Information 3 17

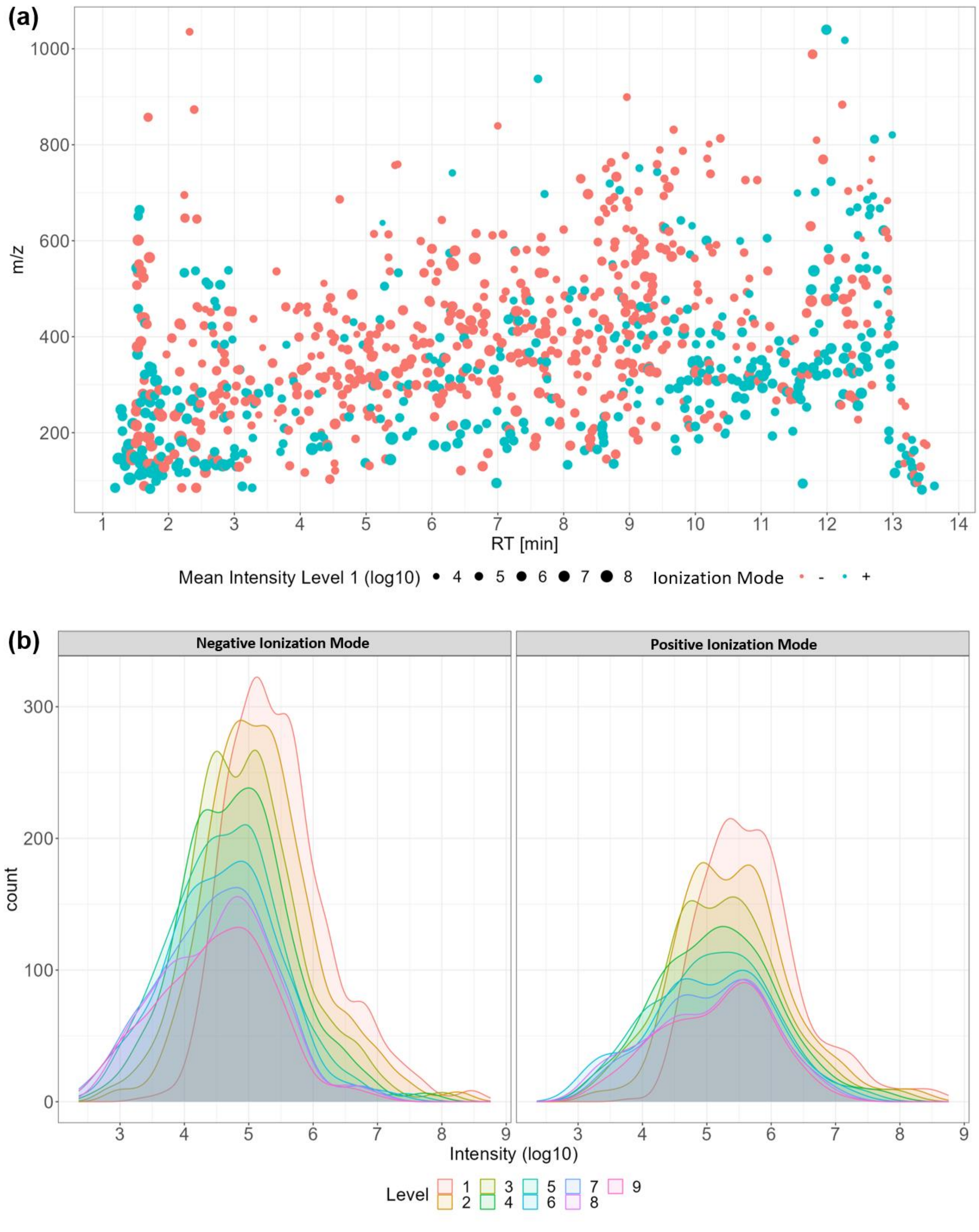

Fig. S1 *(a) Feature plot of all detected features with color according to the ionization mode. (b) Plot for each level showing the distribution of peak intensities of monoisotopic ^{12}C ions separately for the two ionization modes*

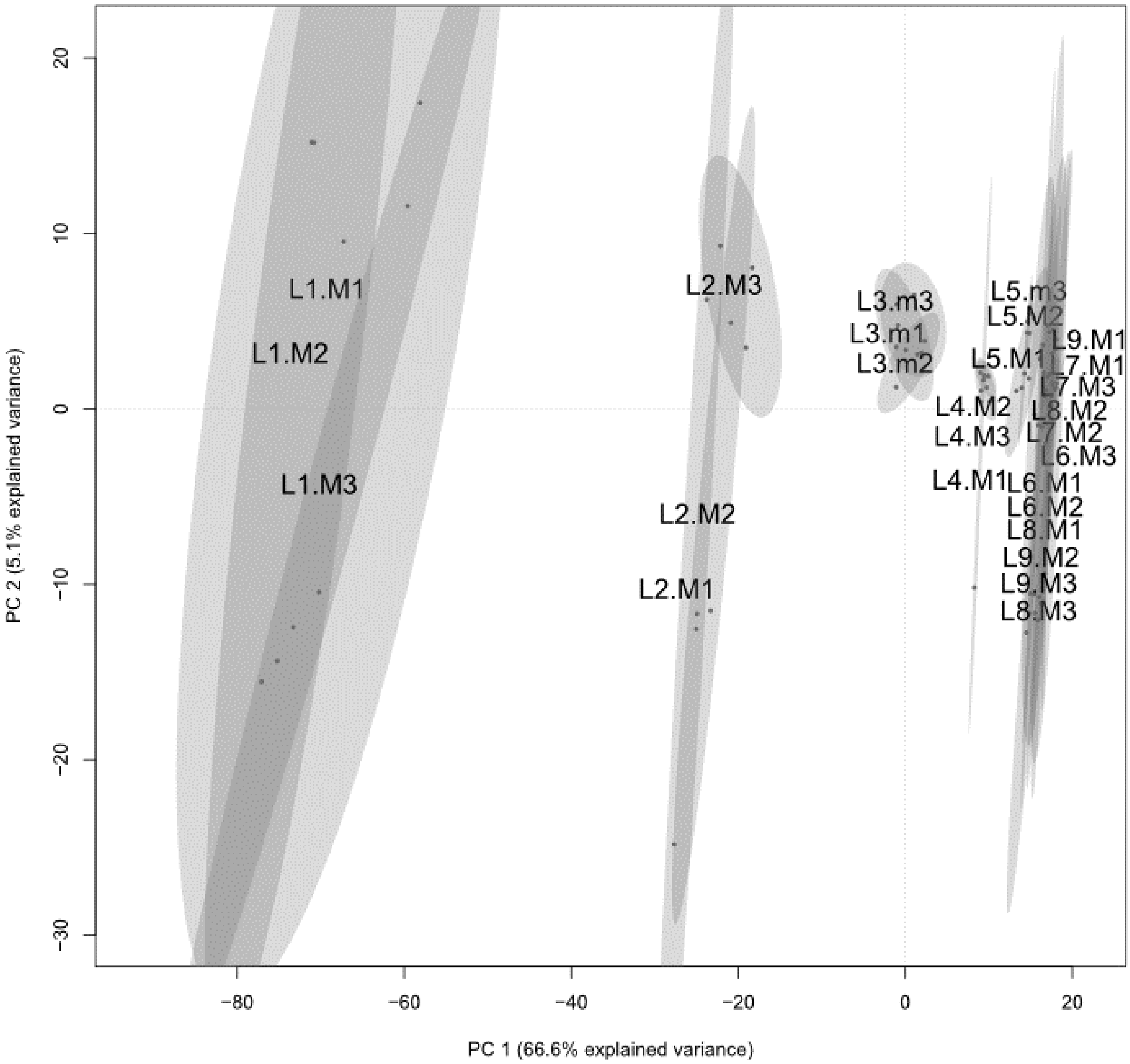

Fig. S2 *Principal component analysis calculated from the abundances of the native features of the internally standardized samples (NL), showing all dilution levels (L1-9), each measured three times (M1-3)*

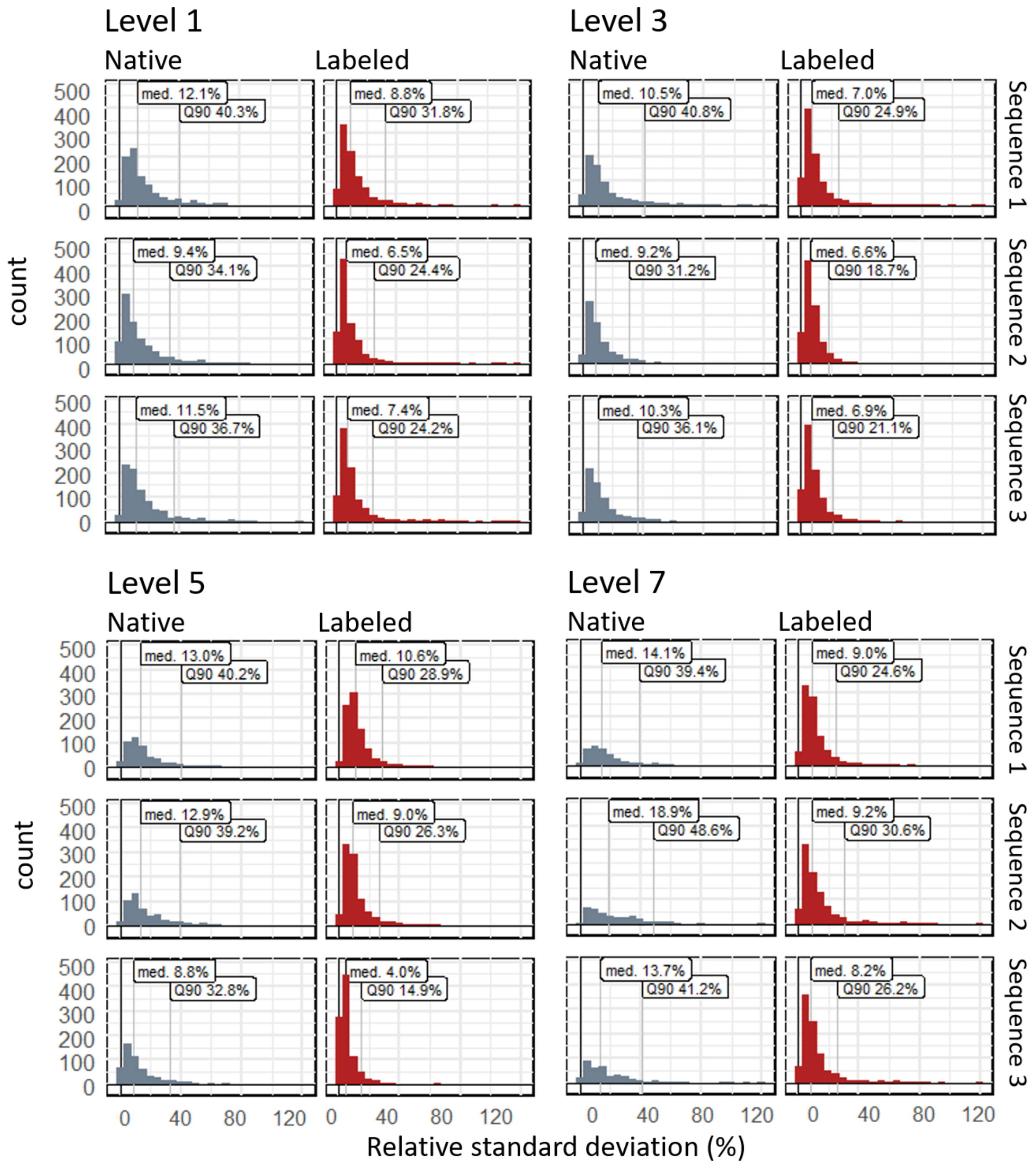

Fig. S3 *Distribution of the relative standard deviations shown for level 1, 3, 5, and 7 of native and labeled features for all three measurement sequences*

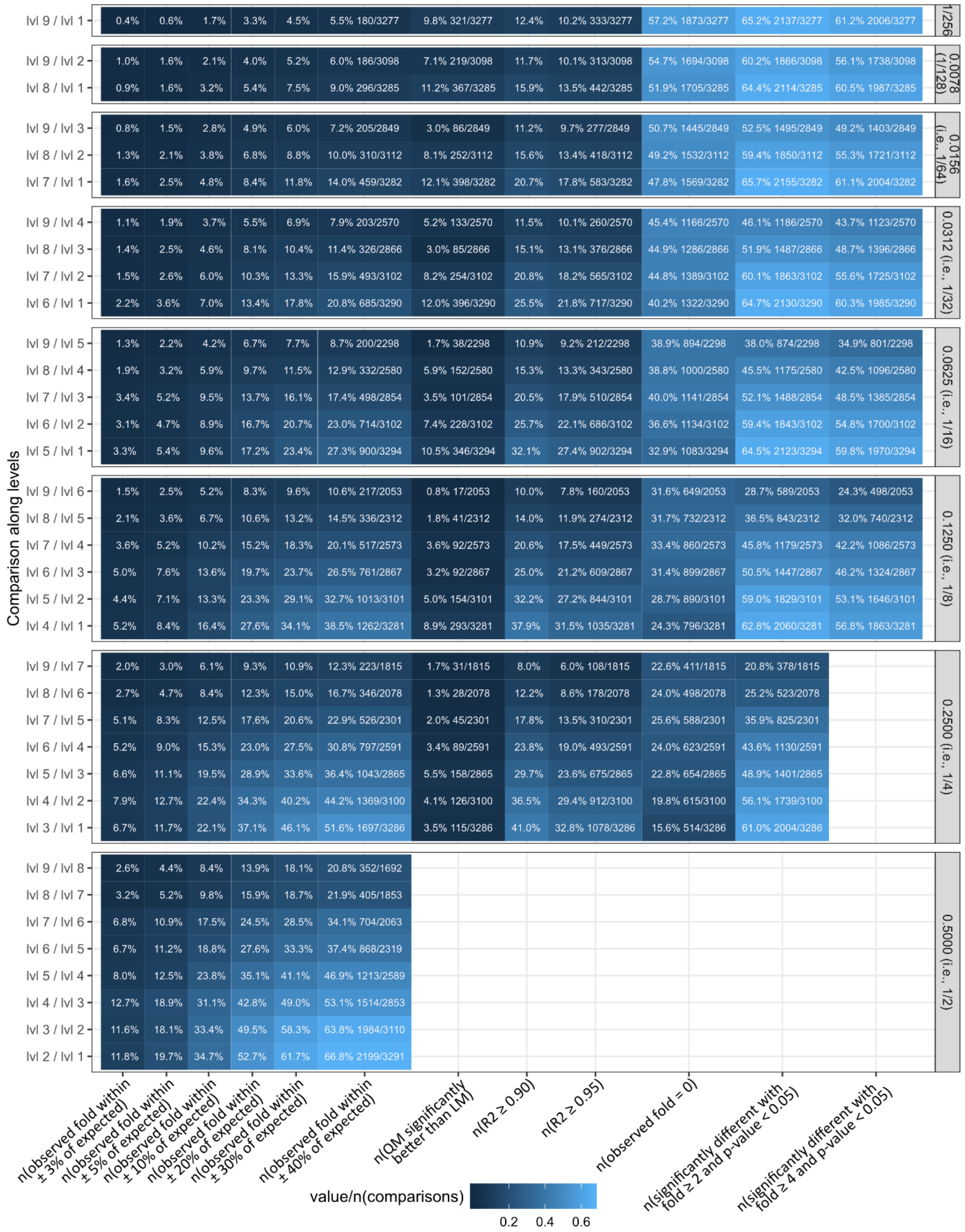

Fig. S4 *Overview of linearity with different metrics*

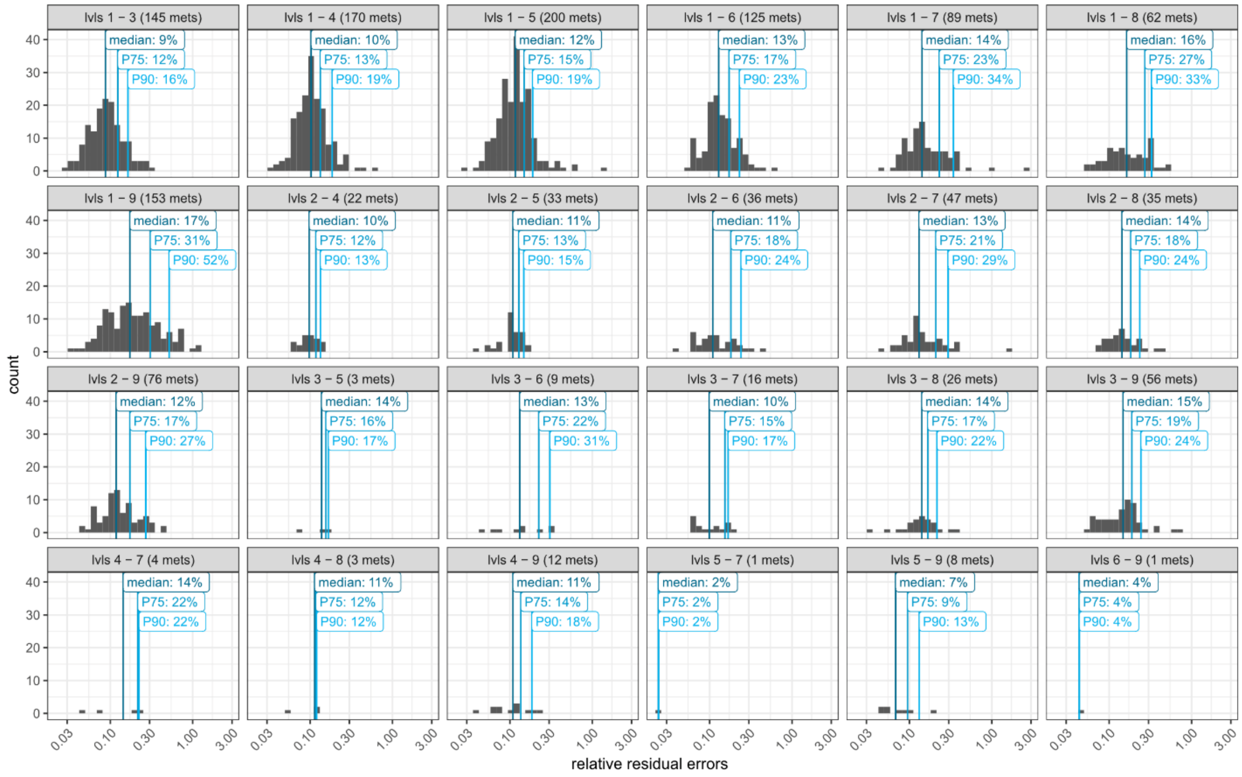

Fig. S5 *Distribution of mean relative residual errors (calculated with (value – predicted)/value). Each metabolite (mets) is only shown in the longest linear range (e.g. a metabolite linear from level 1 to 5 is not shown in level 1 to 3)*

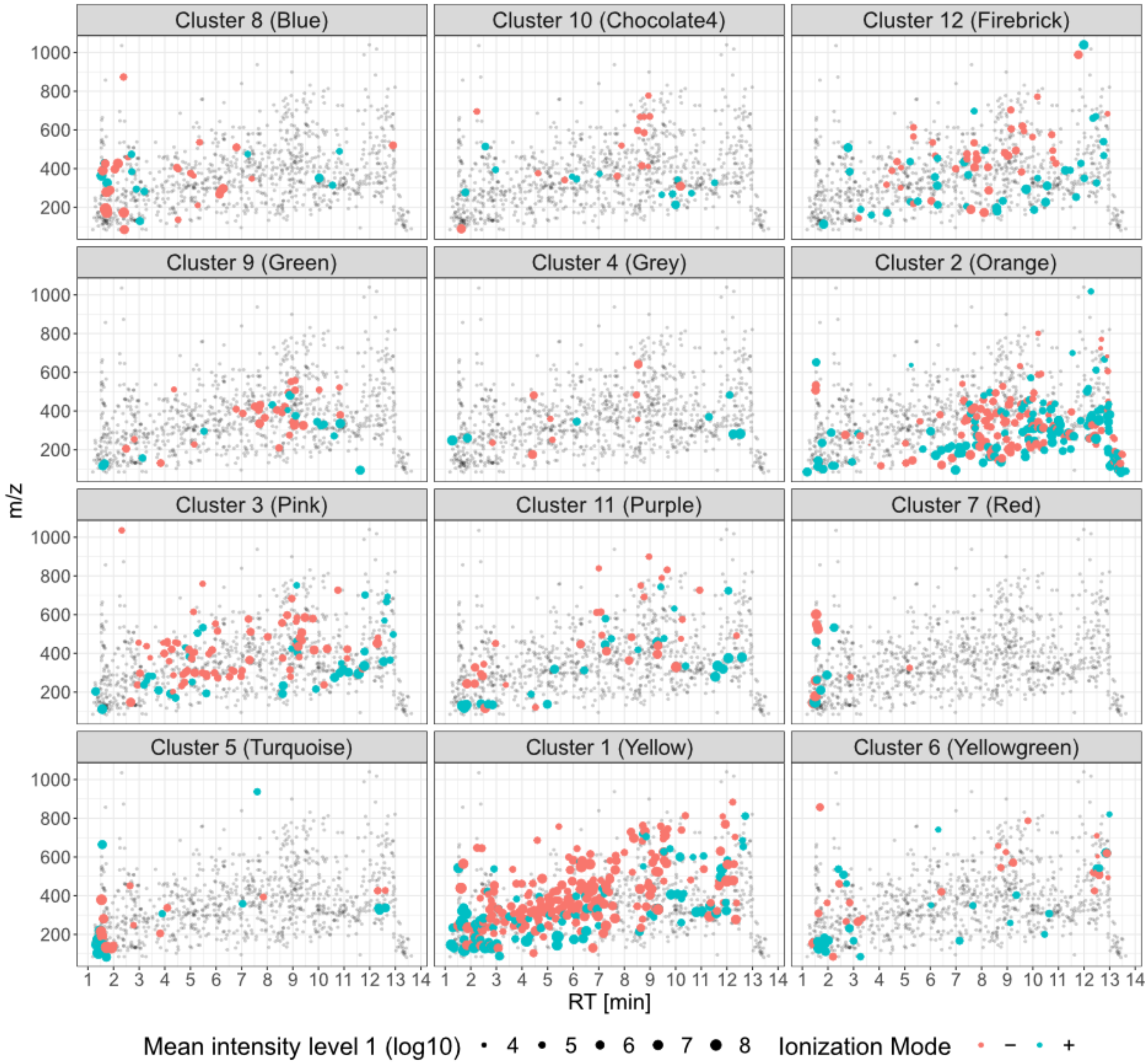

Fig. S6 *Feature plots showing retention time in minutes (RT [min]) versus mass-to-charge ratios (m/z) of features with colors indicating the ionization mode (red and blue dots) for each cluster extracted from the heatmap (Fig. 3). The dot size is proportional to mean intensity (log10-scaled; circle-area) observed in the samples of level 1 for each feature. The grey dots indicate all features regardless of the heatmap cluster and ionization mode*

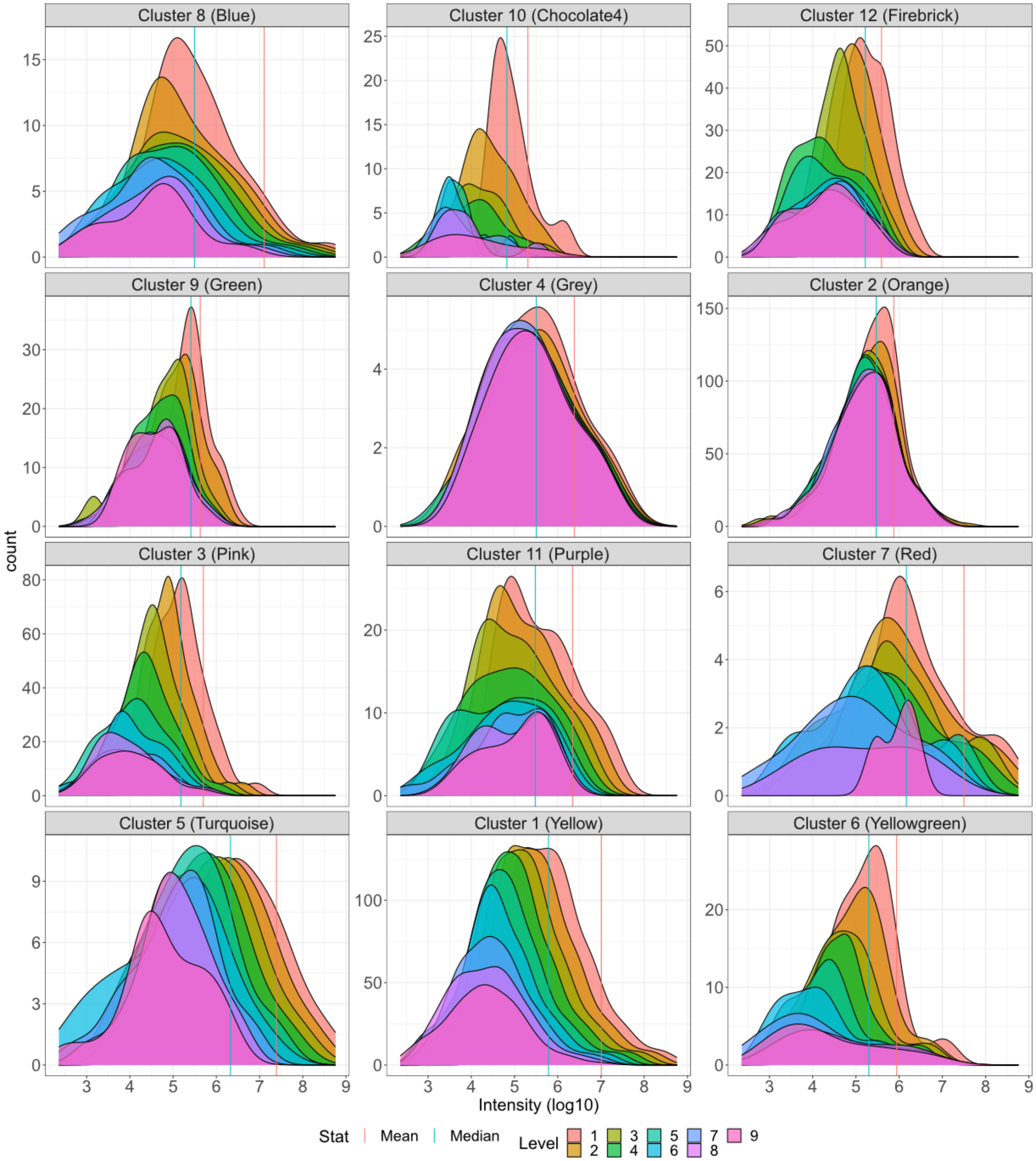

Fig. S7 *Distribution of signal intensity of metabolites of each heatmap cluster and dilution level. Vertical lines indicate mean (red) and median (blue) value of level 1 of the respective heatmap cluster*

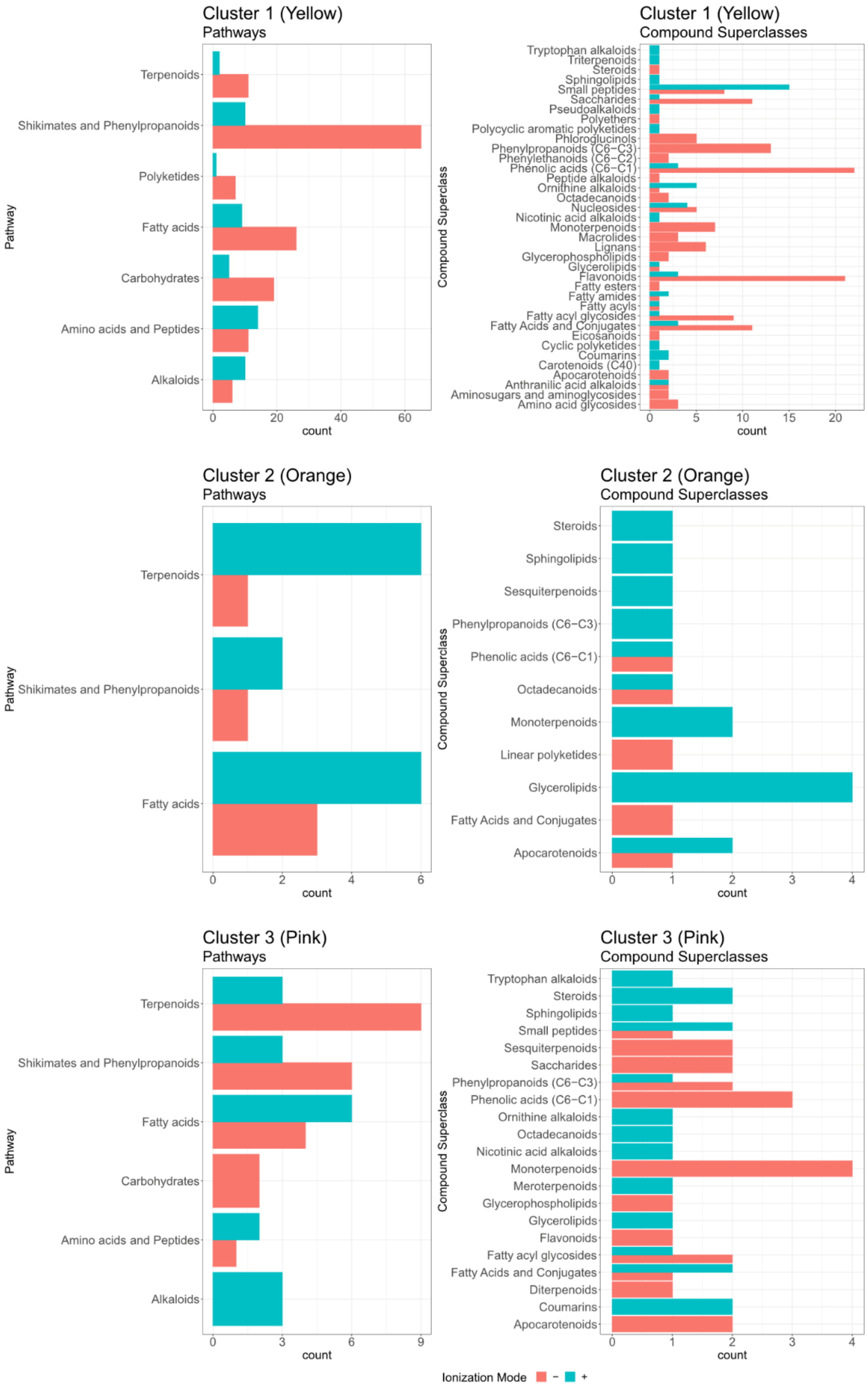

Fig. S8 *Bar charts showing the number of metabolites of the respective NPC pathway (left) or compound superclass (right) for the heatmap clusters cluster 1 (yellow; top), cluster 2 (orange; middle), and cluster 3 (pink; bottom). The color indicates the ionization mode the features' were measured in*

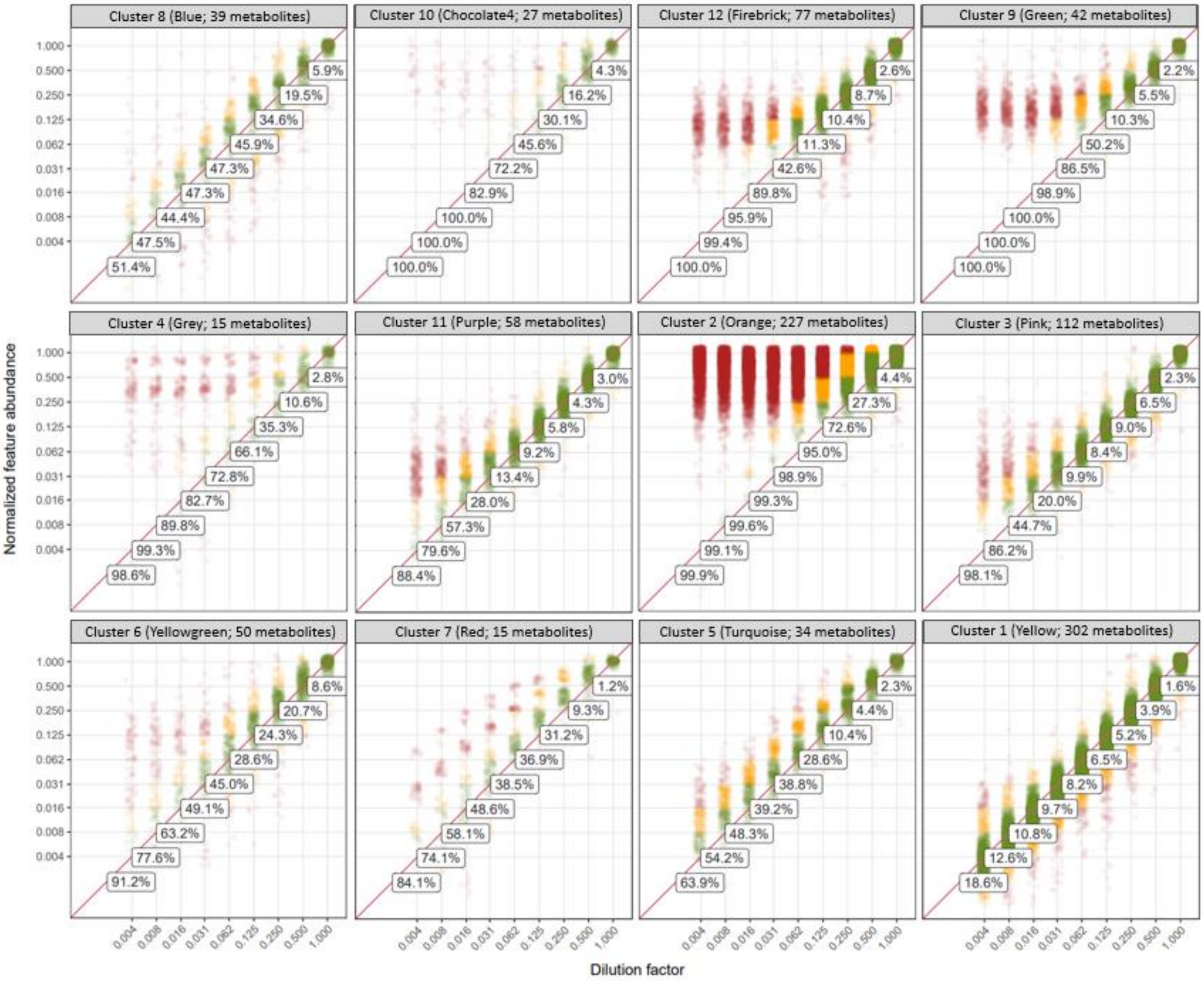

Fig. S9 *Normalized abundances of all metabolites of the respective cluster are shown with observed peak areas versus expected values. Green color indicates metabolites with a fold-change within the 2 or 0.5 interval relative to the expected value, yellow color indicates a fold-change between 2 and 4 or 0.5 and 0.25, and red color indicates a fold-change value outside the yellow area. The labels indicate the percentage of metabolites outside the green interval*

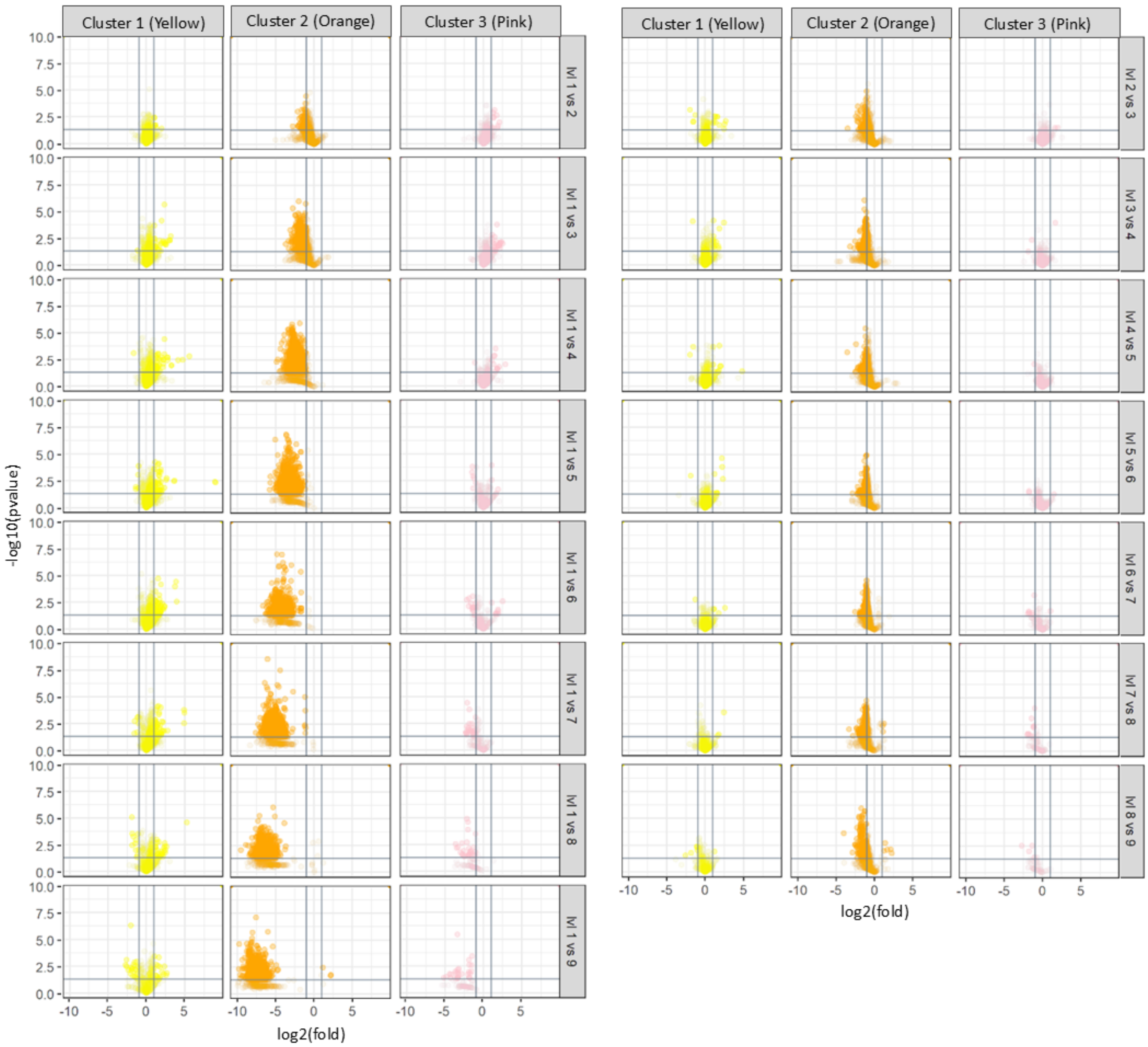

Fig. S10 *The volcano plots show the metabolites of the respective cluster and compare two dilution levels (level 2 to 9 from top to bottom)*

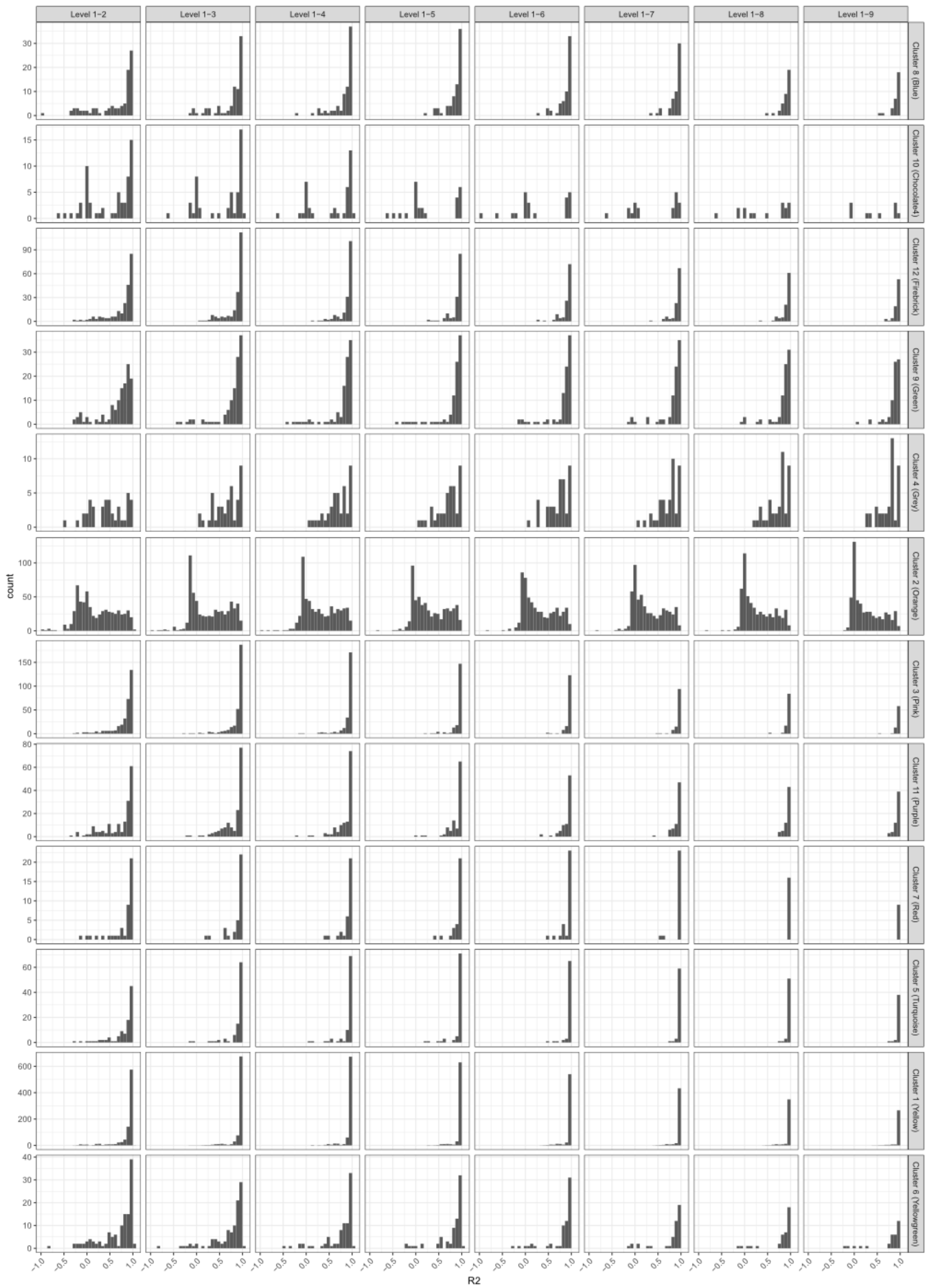

Fig. S11 *Distribution of R2 values obtained from linear models for metabolites of each heatmap cluster. Only specific levels were used for the calculation of the linear models (vertical subplots)*

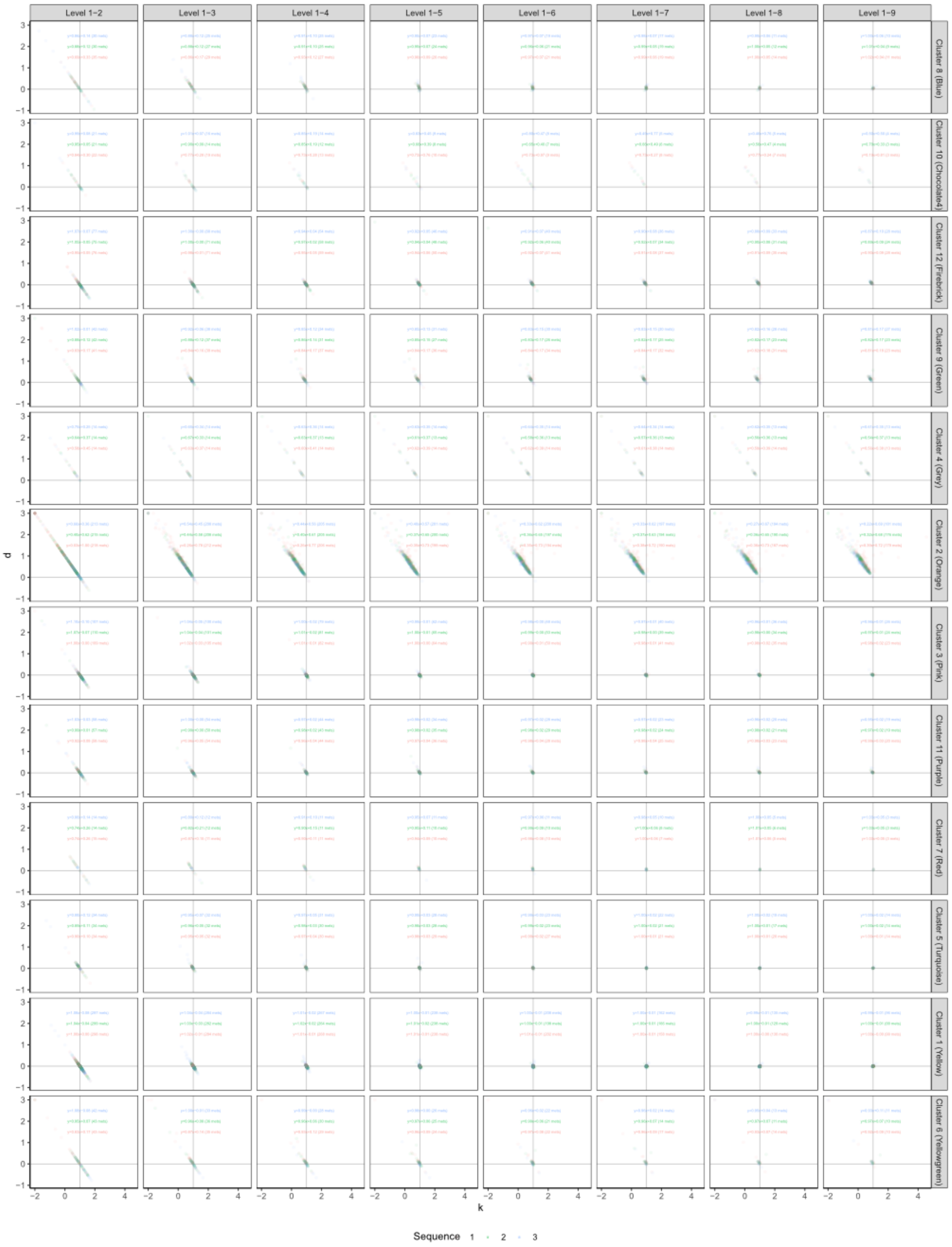

Fig. S12 *Comparison of coefficients k and d of linear models for metabolites of each heatmap cluster. Abundance levels were normalized to the mean of level 1. The three equations inside each subplot indicate the median coefficients of all metabolites of the same sequence (measurement sequences 1-3). Only specific levels were used for the calculation of the linear models (vertical subplots)*

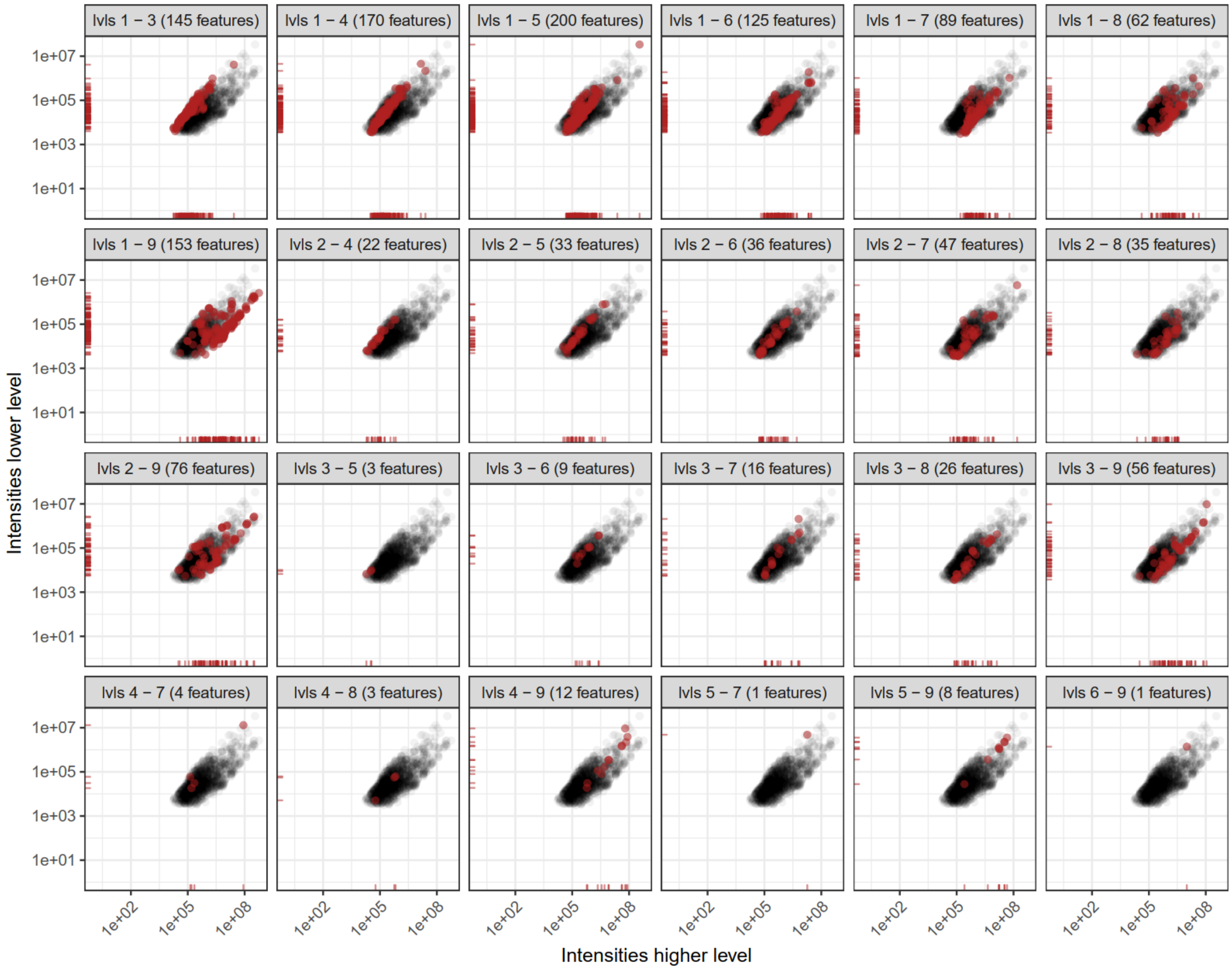

Fig. S13 *Distribution of average signal intensity in higher (x) and lower (y) level. The linear range of a particular metabolite is defined as the maximum dilution level sequence up to which the metabolite had an R2 value of at least 0.95, and a quadratic model was not better. (Thus, for each metabolite the longest sequence of continuous dilution levels was determined, and the respective signal intensity values have been illustrated) Red dots: assigned to the particular dilution level range, black dots: all features*

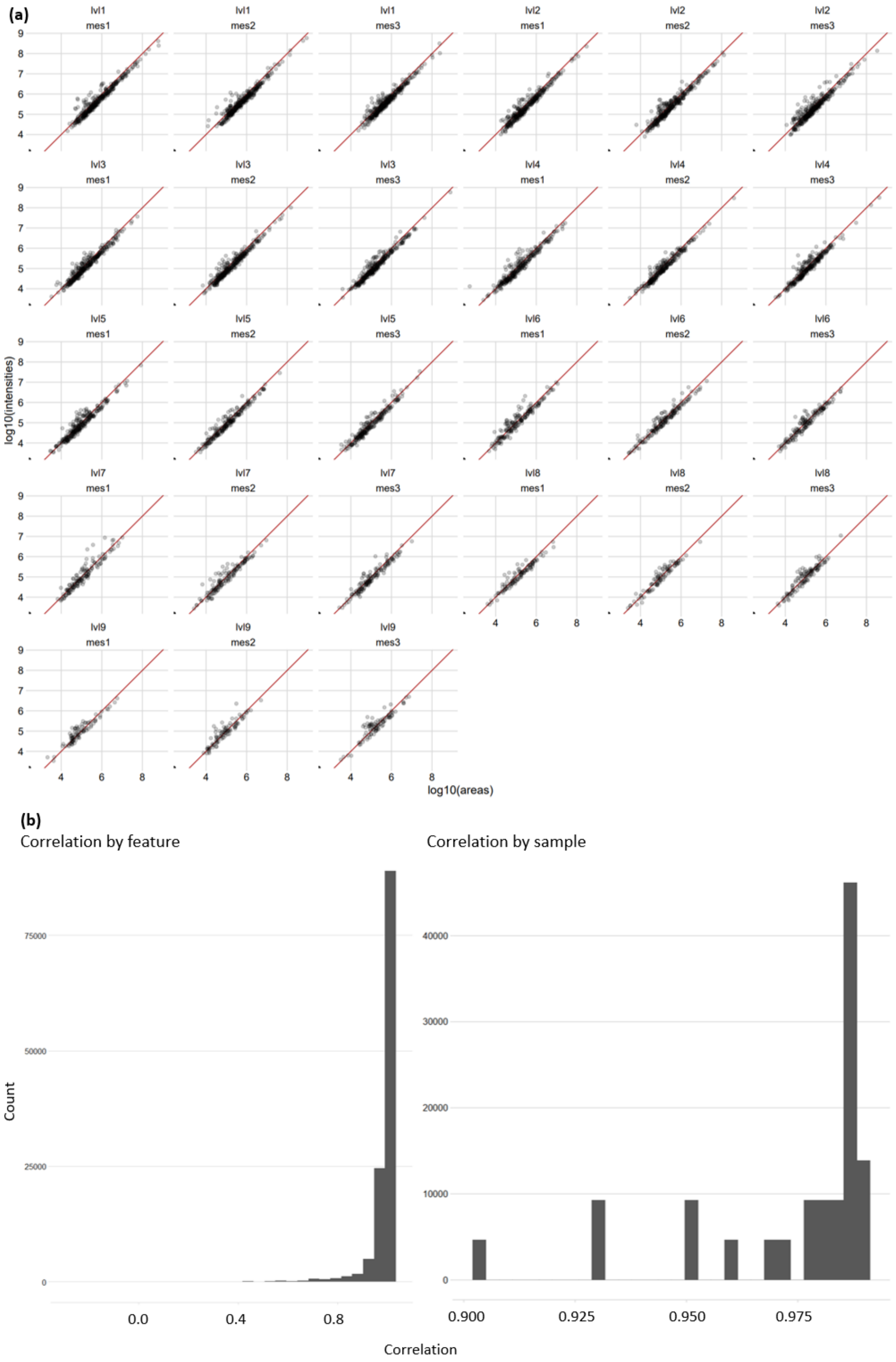

Fig. S14 (a) Correlation of metabolite intensities and abundances for each level and measurement, (b) correlation of area and intensity per sample and feature

Data Processing parameters

Supporting Information 1. Parameters used for raw data evaluation with MetExtract II.

Parameter	Parameter value
isotopic purity native and U-^{13}C-labeled material	98.87% and 98.30%
maximum allowed isotopolog deviation	+/- 15%
maximum allowed mass deviation	+/-3 ppm
scales width	3 – 11
minimum chromatographic peak correlation	0.85
retention time start and end	1 and 15 min
intensity threshold	1E4 units
EIC ppm	+/- 5 ppm
Cn counts to search for	3 – 60
retention time alignment	No
maximum bracketing deviation	10 ppm and 0.1 minutes

Supporting Information 2. Parameters used to obtain MSMS spectra with mzMine3.

Module/Parameter	Parameter value
Mass detection – Noise level	1 x 1E4
ADAP Chromatogram Builder	
m/z tolerance (scan-to-scan)	5 ppm
Smoothing	Savitzky Golay
Local minimum feature resolver	
Min ratio of peak top/edge	1.5
Isotopic peak finder	
Elements	H, C, N, O, S
Maximum charge	2
Feature list rows filter	
Join aligner	
m/z tolerance (sample-to-sample)	10 ppm
Peak finder (multithreaded)	
Retention time tolerance	0.1 min
Duplicate peak filter	
Correlation grouping	
Feature shape correlation	Pearson
Feature height correlation	Pearson
Ion identity networking	
Feature list blank substraction	
Minimum # of detections in blanks	3
Quantification	Area
Fold change increase	500%
Feature list rows filter	Features with MS2 scan

Supporting Information 3. Statistical analysis procedures.

Statistical analysis was carried out in R (https://r-project.org, version 3.5.3).
Either the peak areas of the native, monoisotopic compound forms (N) or those of the isotopologs representing the fully ^{13}C-labeled metabolite ion (L) were used. For internal standardized features the peak areas of these two forms were divided (NL).
A feature must have been detected in at least 3 replicates of at least one experimental group to have been used and only the most abundant feature (ion) per metabolite was used. Missing values were replaced by zero. The data matrix was auto-scaled for multivariate analysis.
For Venn diagrams, a feature must have been detected in at least 3 replicates in order to have been assigned to the respective group.
Principal component analysis was calculated based on auto-scaled peak tables with the ggbiplot package (https://github.com/vqv/ggbiplot).
Heatmap and hierarchical cluster analysis (HCA) analysis was calculated using squared Euclidean distance and ward-linkage.
The Welch Two Sample t-test in combination with a mean-fold-change was used to test for a significant difference. A critical alpha value of 0.05 and a minimum mean-fold change of ≥ 2 or ≤ 0.5 designated the significance thresholds. For features only present in only either of the two compared groups, the mean-fold change is equal to 0 or an infinite value.
A lack of fit test was employed to test if a quadratic model approximates the dilution series better than a linear model. Furthermore, the maximum range of dilution levels a metabolite is linear in was established by full enumeration, meaning that for any two dilution levels and all within those (e.g., level 2 and 5 as well as 3, and 4) a linear model and a lack of fit test was calculated. Then, the maximum linear range for a metabolite was defined as those levels where the linear model resulted in an R2 value of at least 0.95 and the lack of fit test did not report that a quadratic model approximated the abundances better than a linear model.
